# Incorporation of α-MnO_2_ Nanoflowers into Zinc-Terephthalate Metal–Organic Frameworks for High-Performance Asymmetric Supercapacitors

**DOI:** 10.1021/acsomega.2c07808

**Published:** 2023-02-09

**Authors:** Balaji Chettiannan, Arun Kumar Srinivasan, Gowdhaman Arumugam, Shanavas Shajahan, Mohammad Abu Haija, Ramesh Rajendran

**Affiliations:** † Department of Physics, Periyar University, Salem 636011, Tamil Nadu, India; ‡ Department of Chemistry, Khalifa University, P.O. Box, 127788, Abu Dhabi 127788, United Arab Emirates; § Center for Catalysis and Separations, Khalifa University of Science and Technology, P.O. Box, 127788, Abu Dhabi 127788, United Arab Emirates

## Abstract

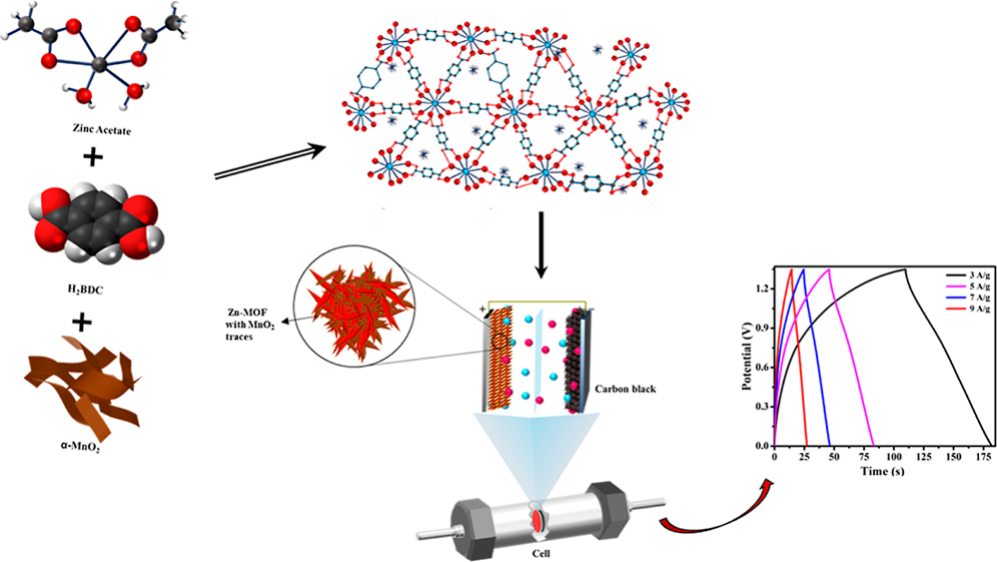

Herein, we report the synthesis of α-MnO_2_ nanoflower-incorporated zinc-terephthalate MOFs (MnO_2_@Zn-MOFs) via the conventional solution phase synthesis technique as an electrode material for supercapacitor applications. The material was characterized by powder-X-ray diffraction, scanning electron microscopy, transmission electron microscopy, and X-ray photoelectron spectroscopy techniques. The prepared electrode material exhibited a specific capacitance of 880.58 F g^–1^ at 5 A g^–1^, which is higher than the pure Zn-BDC (610.83 F g^–1^) and pure α-MnO_2_ (541.69 F g^–1^). Also, it showed a 94% capacitance retention of its initial value after 10,000 cycles at 10 A g^–1^. The improved performance is attributed to the increased number of reactive sites and improved redox activity due to MnO_2_ inclusion. Moreover, an asymmetric supercapacitor assembled using MnO_2_@Zn-MOF as the anode and carbon black as the cathode delivered a specific capacitance of 160 F g^–1^ at 3 A g^–1^ with a high energy density of 40.68 W h kg^–1^ at a power density of 20.24 kW kg^–1^ with an operating potential of 0–1.35 V. The ASC also exhibited a good cycle stability of 90% of its initial capacitance.

## 1 Introduction

In the present century, the exponentially increasing global population and the tumbling non-renewable resources pushed us to utilize consumable renewable energy sources. Though the production of energy from reliable sources can be made possible, the search for proper energy storage systems remains a challenge.^1^ In the midst of traditionally available energy storage systems, supercapacitors sprang up as an alternative to batteries, considering the poor life cycle and feeble power density of batteries.^2^ Despite the fact that batteries have their unique applications, certain areas with the requirement of high-power density and longer life cycles make them inutile. On the other hand, supercapacitors can provide high power and undergo longer charging and discharging cycles. They have been successfully employed in various applications as multiresponsive healable supercapacitors,^3^ piezoelectric-driven self-charging supercapacitors,^4^ and in wearable and portable electronic devices by assembling the supercapacitor as a device with flexible electrodes.^5,6^ Researchers are currently focused on designing electrode materials with different morphologies and larger surface areas to increase their electrochemical performance.^7,8^ However, factors like poor ion diffusion and inadequate structural stability prevent them from being used in certain fields that need high rate and durable energy conversion and storage.^9^ This drawback of poor energy density can be overcome by selecting and properly optimizing electrode materials of the supercapacitors.^10,11^

Lately, MOFs and their derivatives were introduced into the field of energy storage technologies due to their excellent porosity, tunable structure, and stable morphology.^12^ One of the special characteristics of MOFs is their coordinative arrangement and this kind of arrangement is very much suitable for charge transfer and storage; therefore, it can be used as electrode materials in electrochemical supercapacitors.^13,14^ However, most of the MOFs show poor electrical conductivity.^15^ Pristine MOFs in the application of energy storage are typically viewed as insufficient to produce the desired outcomes due to their relatively low intrinsic conductivity.^16^ The electrical conductivity and electrochemical reaction sites of pristine MOFs could be improved by incorporating conductive additives [e.g.,: carbon black (CB), conductive polymers, and so forth.] and metal oxides.^17^ Moreover, the pristine MOF can be used as a sacrificial template to form a pure metal oxide, metal oxide/carbon composite, or carbon, which can be used as electrode materials for supercapacitors.^18,19^

Among the choice of metal oxide additives, MnO_2_ is a promising material due to its abundant availability, lack of toxicity, low cost, several oxidation states, wide voltage window, high surface area, and high theoretical capacitance.^20^ Additionally, MnO_2_ has already exhibited its performance as the primary electrode material for supercapacitors by providing a high capacitance.^21^ It can be used with MOFs to develop a synergistic effect and improve active sites to enhance the overall electrochemical performance. For instance, Huang, Pang, and Lai et al. reported a simple in situ self-transformation method to obtain a MnO_2_@MOF composite material for adaptable energy storage devices. A solid-state flexible SC was fabricated based on prepared MnO_2_@MOFs and activated carbon as positive and negative electrodes, respectively, which exhibited an areal capacitance of 175 mF cm^–2^ at 0.5 mA cm^–2^ and possessed a maximum volumetric energy density of 5.1 mW h cm^–3^ (210 W h kg^–1^).^22^ Liu et al. synthesized a pinecone-like core–shell composite with vertically grown MnO_2_ nanosheet arrays decorated on the MFe_2_O_3_ derived from Fe-MOF. A HSC device was assembled using pinecone-like MFe_2_O_3_@MnO_2_ as the anode and urchin-like NiCo_2_O_4_ as the cathode, and it showed a notable specific capacitance of 804.1 W kg^–1^ in the potential range of 1.1–0.3 V with an energy density of 86.8 W h kg^–1^. The cyclic stability of the device was improved when the MnO_2_ quantity is adjusted to the optimum level.^23^ Pang and Zhu et al. synthesized Ni-HHTP MOFs in situ grown on the surface of MnO_2_. The composite material considerably increased the conductivity and supplied ample ion transport routes. Additionally, the assembled aqueous asymmetric supercapacitor (ASC) with MnO_2_@Ni-HHTP as the anode and activated carbon as the cathode showed a specific capacitance of 368.2 F g^–1^ at 1 A g^–1^ with a high energy density of 35.8 W h kg^–1^ at a power density of 600 W kg^–1^ with a capacitance retention of 87.4 percent at 3000 W kg^–1^. Even after 3000 cycles, the assembled device still displayed a high coulombic efficiency of 95.4% and a capacitance of 233.1 F g^–1^.^24^ Huang and Shi et al. reported novel electrochromic MOF-based hierarchical self-assembled nanosheets (EV-HNSs) as the negative electrode for supercapacitors which are synthesized using negative electroactive organic viologen ligands and europium ions via a dual-template-directed approach. The obtained self-assembled nanosheets (EV-HNSs) showed an areal capacity of 186.25 mF cm^–2^ at 1 mA cm^–2^ in a potential window of −0.9 to −0.1 V (vs Ag/AgCl). Further, an ASC device was fabricated using electrodeposited MnO_2_ as the positive electrode, and EV-HNSs as the negative electrode. The ASC exhibited a high areal energy density of 9.4 μW h cm^–2^ at 775 μW cm^–2^ in an operating potential of 1.55 V.^25^ Chen, Xu, and Jiao et al. synthesized a porous NiO-incorporated Ni-MOF/NF with a cylindrical cage-like structure to promote the transport of electrons and ions in the electrochemical energy storage process. The prepared NiO@Ni-MOF/NF delivered a high specific capacity of 1853 C cm^–2^ at 1 mA cm^–2^. An HSC was constructed using NiO@Ni-MOF/NF as the anode and CNT as the cathode, respectively, which delivered a specific capacitance of 144 F g^–1^ at 1 Ag^–1^ with an energy density of 39.2 W h kg^–1^ at a power density of 7000 W kg^–1^. It also displayed good cycling stability with 94% capacity retention after 3000 cycles.^26^ Yang and Zhao et al. prepared core–shell MnO_2_ nanotubes@nickel–cobalt–zinc hydroxide (NiCoZn–OH) nanosheets using MOFs as a template. The prepared MnO_2_@NiCoZn–OH electrode showed a specific capacitance of 1569.1 F g^–1^ at 1 A g^–1^ and a high rate performance of 54% retention at 30 A g^–1^. An ASC device fabricated using MnO_2_@NiCoZn–OH and AC as positive and negative electrodes, respectively, delivered a superior capacitance of 130.7 F g^–1^ at 1 A g^–1^ with a high energy density of 49.4 W h kg^–1^ at 842.7 W kg^–1^, and an excellent capacitance retention of 91.3% after 10,000 cycles.^27^

In this work, we present a composite made of Zn-BDC and MnO_2_ (MnO_2_@Zn-MOF) serving as the electrode material for the supercapacitor. First, we use a hydrothermal approach to synthesize α-MnO_2_ nanoparticles. This synthesized α-MnO_2_ is then in situ incorporated into the zinc-terephthalate MOF (Zn-BDC) using a simple solution phase synthesis technique. Here, α-MnO_2_ is selected owing to its superior electrocatalytic ability compared with β-MnO_2_, ϵ-MnO_2_, and γ-MnO_2_ phases reported in the previous literature study.^28^ The MnO_2_@Zn-MOF nanostructure as electrode materials displayed a specific capacitance of 880.58 F g^–1^ at 5 A g^–1^ and a life cycling performance of 93% retention after 10,000 cycles at a current density of 10 A g^–1^ with a potential range of 0–0.6 V. The composite electrode material demonstrated a significantly improved performance due to its thin rod-like morphology with enhanced reactive sites and redox-active manganese oxide traces. The ASC device constructed using the as-prepared material as the anode and commercial CB as the cathode delivered a specific capacitance of 160 F g^–1^ at 3 A g^–1^ with a high energy density of 40.68 W h kg^–1^ at a power density of 2024 W kg^–1^. The device also retained 90% of the initial capacitance after 10,000 cycles at 9 A g^–1^.

## 2 Experimental Section

### 2.1 Materials

Potassium permanganate (99%, Sigma-Aldrich), zinc acetate dihydrate (99%, Sigma-Aldrich), terephthalic acid (BDC) (98%, Sigma-Aldrich), triethanolamine (99%, Molychem), dimethyl formamide (DMF) (99.5%, Molychem), and hydrochloric acid (36.5%, Molychem) were used as received without any further purifications. Double-distilled deionized water was used for all the electrochemical studies.

### 2.2 Synthesis of α-MnO_2_ Nanoflowers

The α-MnO_2_ nanoflowers were synthesized via a typical hydrothermal method. In this preparation, 1.5 mmol KMnO_4_ and 5 mmol concentrated HCl were added to 15 mL of deionized water. The mixture was stirred vigorously for several minutes to form a transparent purple solution and then it is transferred into a 50 mL Teflon-lined stainless-steel autoclave, and the autoclave was sealed and heated at 140 °C in a hot air oven for 15 h to obtain the precipitate. The precipitates were cooled down to room temperature, collected by centrifugation, and washed several times with distilled water. The final product was obtained by drying at 60 °C in a hot-air oven overnight.

### 2.3 Preparation of Zn-MOF

In the typical synthesis, 1.5 g of terephthalic acid (BDC) (organic linker) and 4.1 g of zinc acetate dihydrate [Zn(CH_3_COO)_2_·2H_2_O] (metal precursor) were simultaneously dissolved in a 150 mL DMF solution. Then, 3 mL of triethanolamine was gradually added into the mixture under continuous magnetic stirring at room temperature and the stirring was continued for 36 h. Finally, the brownish-white precipitates were obtained. After centrifuging, the sample was washed several times simultaneously with ethanol, distilled water, and DMF. The final products were dried at 60 °C overnight in a hot-air oven and stored for further characterization. The final product, Zn-MOF, is named ZM, for our convenience.

### 2.4 Incorporation of α-MnO_2_ Nanoflowers into Zn-MOFs

Twenty milligram of the as-synthesized α-MnO_2_ was added to 40 mL DMF, and this suspension was gradually added to 150 mL DMF solution containing 4.1 g zinc acetate dihydrate [Zn(CH_3_COO)_2_·2H_2_O] and 1.5 g of terephthalic acid (BDC) under steady magnetic stirring. The brownish precipitates were formed upon the dropwise addition of 3 mL triethanolamine in the above mixture under constant magnetic stirring at room temperature for 36 h. Further, the precipitates were collected by centrifugation and washed several times with ethanol, distilled water, and DMF. The final products were dried at 60 °C overnight in a hot-air oven. Then, dried precipitates were stored for further characterization. The final product, MnO_2_@Zn-MOF, is named MZM, for our convenience. The same procedure was used to synthesize MnO_2_@Zn-MOF with different quantities of MnO_2_ (10, 30, and 40 mg) and named M-10, M-30, and M-40, respectively, for our convenience.

### 2.5 Material Characterizations

The crystal structure of the prepared electrode materials was examined by employing powder X-ray diffraction (XRD, Rigaku D/Max ultima3i) using Cu K-α radiation (=0.1542 nm). Field-emission scanning electron microscopy (FE-SEM, Zeiss JSM-7500F) and transmission electron microscopy (TEM, JOEL JEM 2100F) were used to image the morphological features of the produced samples. Energy-dispersive spectroscopy (EDS, Zeiss JSM-7500F) is used for chemical characterization/elemental analysis of materials. X-ray photoelectron spectroscopy (XPS, ULVAC-PHI 5000 Versa probe III) was used to examine the elemental composition and valence states of the materials.

### 2.6 Electrochemical Measurements

An electrochemical workstation (BioLogic SP-150) was used to investigate electrochemical characteristics. The three-electrode configuration was used for all electrochemical experiments, with the prepared electrode serving as the working electrode, a Pt counter electrode serving as the cathode, Hg/HgO serving as the reference electrode, and an aqueous electrolyte solution containing 3 M KOH as the electrolyte. Cyclic voltammetry (CV) and galvanostatic charge–discharge (GCD) studies were performed at different scan rates (5 to 80 mV s^–1^) and current densities (5 to 30 A g^–1^), respectively. Electrochemical impedance spectroscopy (EIS) measurements were recorded with an applied frequency range of 0.01 Hz to 100 kHz.

To prepare the anode, the as-prepared material (MZM), CB, and polyvinylidene fluoride (PVDF) at a weight ratio of 7:2:1 were mixed with a *N*-methyl proline (NMP) solution to form a slurry. The slurry was gently coated on pre-cleaned Ni foam (NF) (1 cm^2^) and dried in a hot-air oven at 50° C for 12 h.

The specific capacitance of electrode materials will be calculated by using the known values of current density *I* (A g^–1^), discharge time Δ*t* (s), the mass of the active materials *m* (mg), and the potential window range Δ*V* (V)^29^
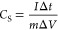
1where *I* is the current density (A g^–1^), Δ*t* is the discharge time (s), *m* is the mass of active material (mg), and *ΔV* is a potential window (V).

### 2.7 Construction of ASC

The electrodes were fabricated as specified in the three-electrode system with a disc-shaped NF with a 1 cm radius and it was used in a two-electrode configuration. The conventional method was used to create the PVA/KOH gel electrolyte.^30^ The pre-made PVA/KOH hydrogel was applied to the Whatman filter paper during the ASC construction process. The two thin stainless-steel discs (current collector) of a Swagelok-type cell were then sandwiched with our sample MZM/NF and carbon-black/NF. BioLogic SP-150 equipment was then used to conduct electrochemical tests. Using CV, GCD, and EIS techniques, the electrochemical characteristics of ASC were investigated. The following relationships were used to compute the specific capacitance (*C*, F g^–1^), energy density (*E*_D_, W h kg ^–1^), and power density (*P*_D_, W kg^–1^) of the ASC device.^31,32^
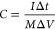
2
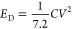
3
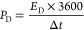
4where *V* is the operating potential (V), *M* is the total mass of the active materials (mg) and Δ*t* is the discharge time (s).

## 3 Results and Discussion

**Figure 1:**
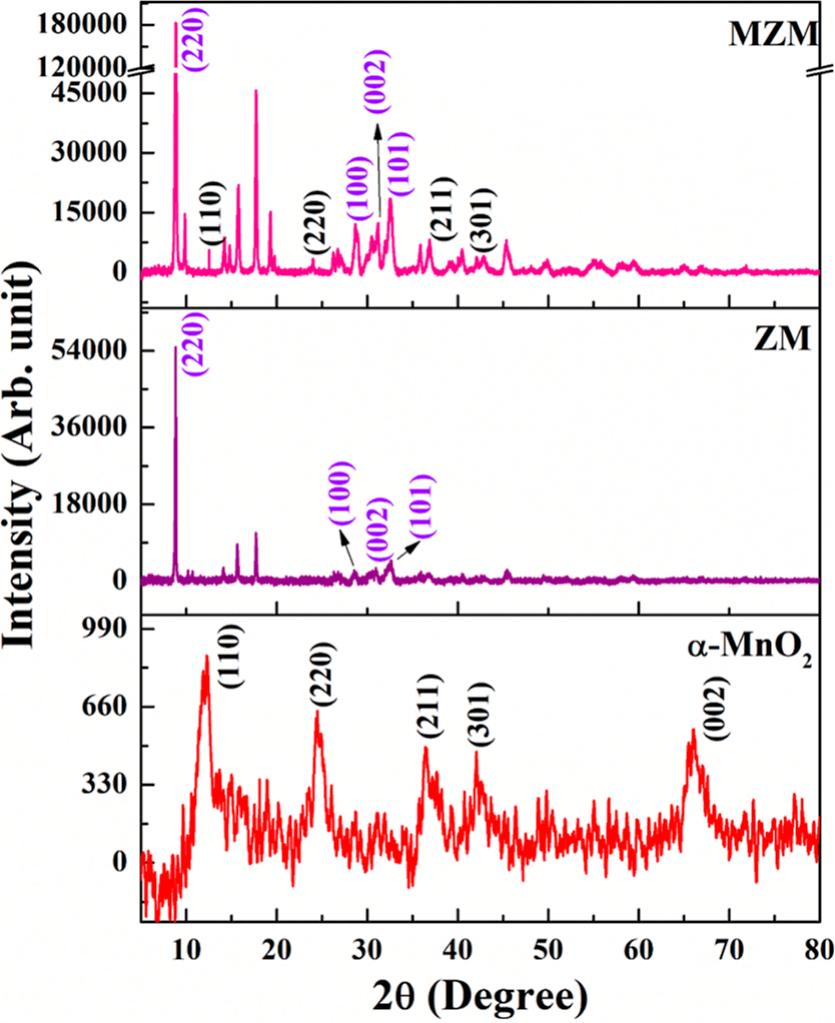
Powder XRD patterns of α-MnO_2_, ZM, and MZM electrode materials.

The XRD patterns of the MnO_2_, ZM, and MZM electrode materials are depicted in Figure 1. The XRD pattern of α-MnO_2_ shows a tetragonal structure and matches well with JCPDS no. 44-0141. The peaks at 12.5, 25.7, 37.5, 42.1, and 65.10 correspond to (110), (220), (211), (301), and (002) crystal planes of α-MnO_2_, respectively.^33^ The XRD patterns of ZM and MZM electrode materials show a cubic crystal structure and are well-matched with CCDC no. 256965. The strong XRD peak at θ = 9.8° demonstrates the growth of crystals in the (220) crystal plane.^34,35^ The additional peaks that appear at 31.10, 33.23, and 35.97, which corresponds to (100), (002), and (101) crystal planes (JCPDS no. 36-1451), are due to the presence of free ZnO clusters with a wurtzite structure.^36^ In MZM electrode materials, the corresponding peaks of α-MnO_2_ were presented in the original position indicating no phase change. The relative intensity of the peak at θ = 9.8 is very high for MZM when compared with the ZM electrode material, which indicates the high crystalline nature of the material. Here, the α-MnO_2_ acted as an auxiliary reagent to mediate the synthesis process reducing the agglomeration as well as enriching the structural defects of the MOFs resulting in high crystallinity.^37−39^ The relative intensity of α-MnO_2_ is significantly lower than the intensity of MOFs, thus indicating the presence of the trace amounts of α-MnO_2_ in the MZM electrode material. Also, the XRD patterns of different quantities of MnO_2_-incorporated Zn-MOF (shown in the Figure S5) indicate that increasing the amount of MnO_2_ to a certain extent, i.e., 20 mg show MOFs with a sharp intense peak at θ = 9.8° and by increasing the quantity of MnO_2_ (30 and 40 mg), the peak intensity was decreased. It can be inferred that 20 mg incorporation is the optimal quantity to obtain high crystallinity.

**Figure 2:**
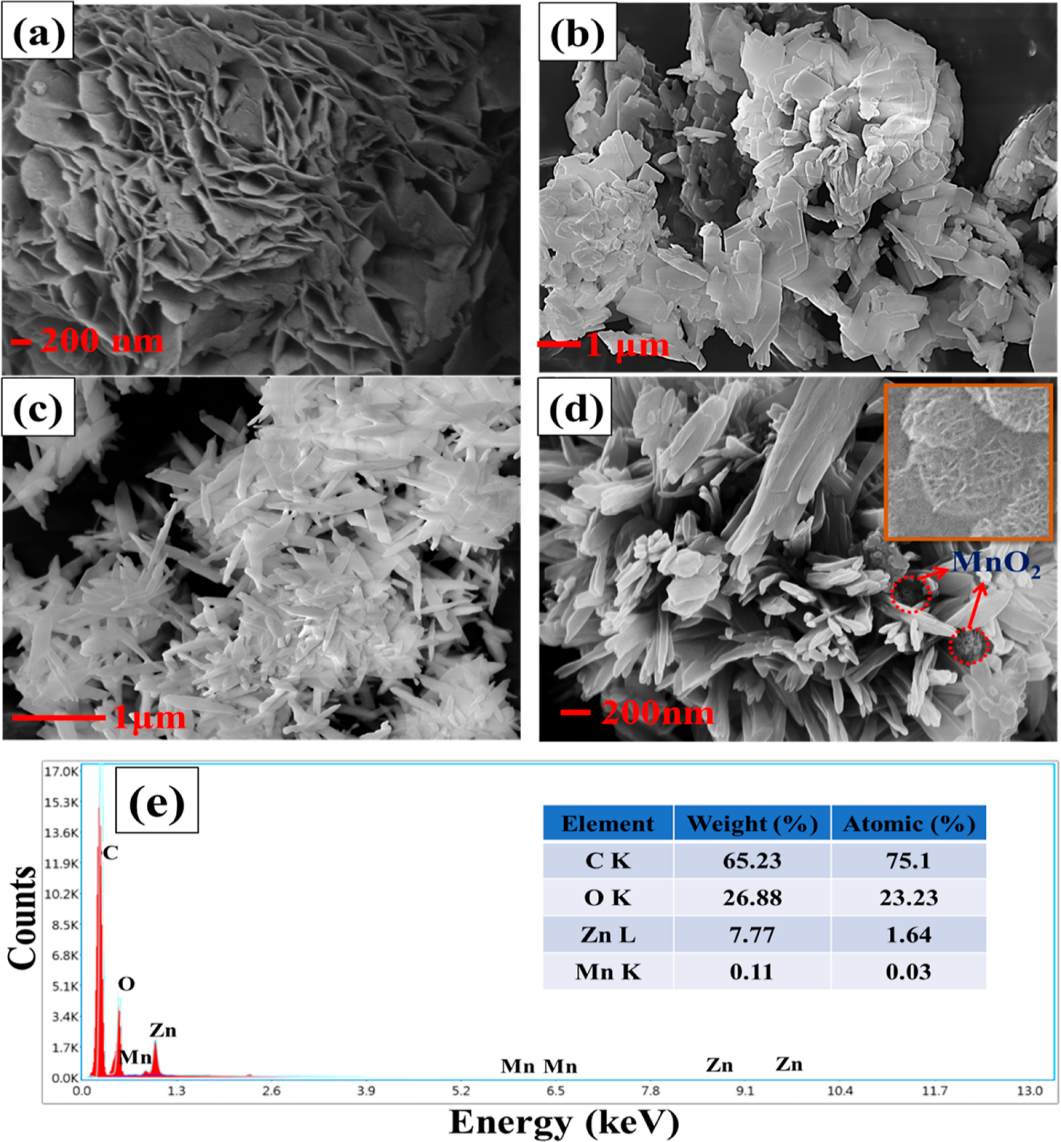
Field-emission scanning electron micrograph of (a) α-MnO_2_, (b) ZM, and (c,d) MZM electrode materials and (e) corresponding EDX spectrum of MZM electrode material.

The surface morphologies of the prepared electrode materials were investigated by FE-SEM analysis. Figure 2a–d shows the FE-SEM images of α-MnO_2_, ZM, and MZM electrode materials, respectively. Figure 2a shows the flower-like morphology of the pure α-MnO_2_. The irregular flake-like morphology was exhibited by the ZM electrode material (Figure 2b). Besides, the MZM material exhibited an inhomogeneous thin rod-like morphology, as shown in Figure 2c,d. This change in the morphology and size of the MOF can be attributed to the addition of α-MnO_2_, which has already been reported in previously reported works in the literature.^17,38,40^ This thin rod-like morphology with comparatively smaller particles may be helpful in providing more metal-active centers for electrochemical reactions. Moreover, a few flower-like structures can be seen in the FE-SEM of MZM, which is indicative of the presence of α-MnO_2_ as this appears to be exactly the same as the SEM images of pure α-MnO_2_ (inset Figure 2d). The EDX spectrum analysis of the MZM electrode material is shown in Figure 2e, respectively. The weight and atomic percentage of the elements present in the MZM electrode material are tabulated in the inset of Figure 2e. It can be inferred that the element Mn is present in the MZM electrode material with a weight percentage of 0.11 and an atomic percentage of 0.03. This confirms the presence of the trace amount of MnO_2_ in the MZM electrode. This aligns well with the XRD results of MZM with relatively low-intensity MnO_2_ peaks.

**Figure 3:**
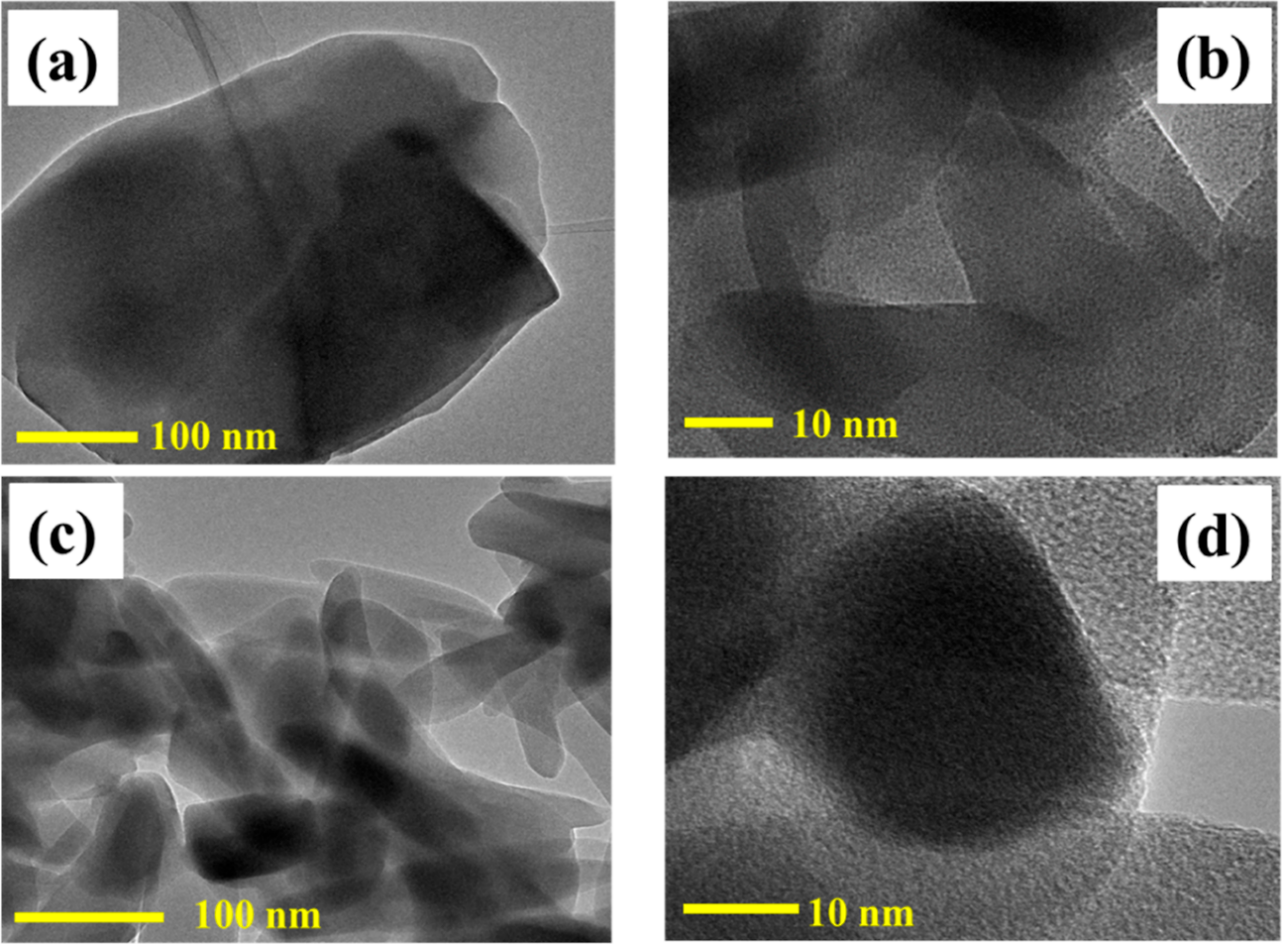
(a,b) Transmission electron micrograph (TEM) of ZM and (c,d) MZM electrode materials.

The transmission electron micrographs (TEM) of the ZM and MZM electrode materials are shown in Figure 3a,b and 3c,d, respectively. The flake-like morphology was obtained for the ZM electrode material and the thin rod-like morphology can be seen for the MZM electrode material and are well consistent with the FE-SEM images. The TEM micrographs of ZM and MZM samples shown in Figure 3b,d at 10 nm demonstrate a wide dispersion of zinc metal nanoparticles as evenly spaced-out black dots in the solid matrix as reported in a previous literature for zinc-based MOFs.^41^ Metal nanoparticle dots appear to be identical. In the TEM micrograph of MZM, α-MnO_2_ could not be found in the focused area which might be due to the presence of only a trace amount of α-MnO_2_.

**Figure 4:**
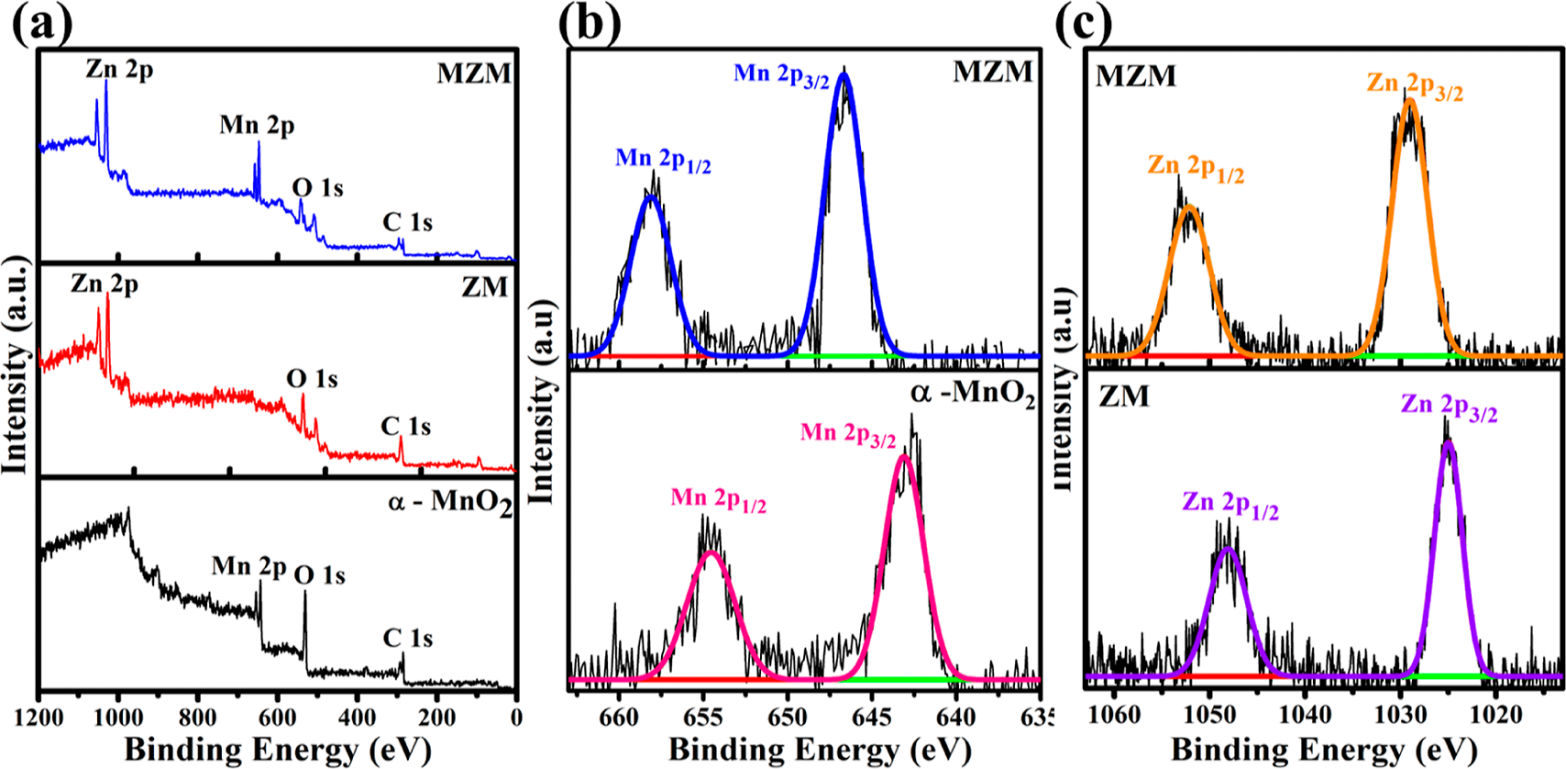
XPS analysis of the electrode materials (a) survey spectrum of α-MnO_2_, ZM, and MZM, (b) high-resolution spectrum of Mn 2p of MZM and α-MnO_2_, and (c) high-resolution spectrum of Zn 2p of MZM and ZM.

The chemical compositions of α-MnO_2_, ZM, and MZM electrode materials were shown by XPS (Figure 4). Figure 4a shows the survey spectra of the prepared materials which indicate that the elements Mn, O, and C exist in α-MnO_2_, Zn, C, and O exist in ZM, and Zn, C, Mn, and O exist in MZM. Mn 2p XPS spectra of MZM and α-MnO_2_ are shown in the Figure 4b. In the MZM electrode material, the Mn 2p spectrum was split into two peaks, which indicate 645.8 eV (Mn 2p_3/2_) and 657.4 eV (Mn 2p_1/2_) binding energies. Similarly, in the pure α-MnO_2_, Mn 2p was split into Mn 2p_3/2_ and Mn 2p_1/2_ with binding energies of 643.7 and 655.2 eV. In both MZM and α-MnO_2_, the spin-energy separation of Mn 2p_3/2_ and Mn 2p_1/2_ is 11.6 eV, which indicates the presence of MnO_2_ species with an Mn^4+^ oxidation state.^42−44^ Moreover, the absence of a satellite peak between Mn 2p_3/2_ and Mn 2p_1/2_ further confirms that the formed compound is MnO_2_ and not MnO.^45^ As shown in Figure 4c, Zn 2p XPS spectra of ZM had two characteristic peaks at 1046.5 and 1024.2 eV attributed to Zn 2p_1/2_ and Zn 2p_3/2_, respectively, revealing the formation of Zn–O bonds between the metal cluster and the linker. Similarly, two characteristic peaks of Zn 2p spectra of MZM at 1050.6 and 1027.5 eV could be attributed to Zn 2p1/2 and Zn 2p3/2, respectively. The spin–orbit doublet peaks of Zn 2p were separated by a binding energy difference of 22.7 eV, which confirms the bivalent valence of the Zn ion. The XPS signal of O 1s of MnO_2_ (Figure S1a) was split into three peaks at 530.5, 531.8, and 532.9 eV, and they were assigned to the Mn–O–Mn bond of the tetravalent metal oxide, Mn–OH bond, and to physically and chemically surface adsorbed water molecules, respectively.^46,47^ The XPS signal of O 1s of ZM (Figure S1b) was split into three peaks at 529.5 eV (O–C=O), 530.9 eV (Zn–O), and 532.3 eV (C=O).^36,42,48^ The XPS signal of O 1s of MZM (Figure S1c) was split into three peaks at 529.8 eV (O–C=O/Mn–O–Mn), 530.5 eV (Zn–O/Mn–O–H), and 532.1 eV (C=O), which are ascribed to the oxygen bonding with Zn and Mn metal clusters and also to the adsorbed water molecule on the surface both chemically and physically.^49−51^ Further, it is obvious that all of the Zn 2p peaks (Figure 4b) and Mn 2p peaks (Figure 4c) of MZM shifted in the direction of increasing binding energies. This can be attributed to the interaction between Zn and Mn ions, moving the 2p peaks in the direction of the more positive binding energies.^52^

### 3.1 Electrochemical Analysis of ZM and MZM Electrode Materials

**Figure 5:**
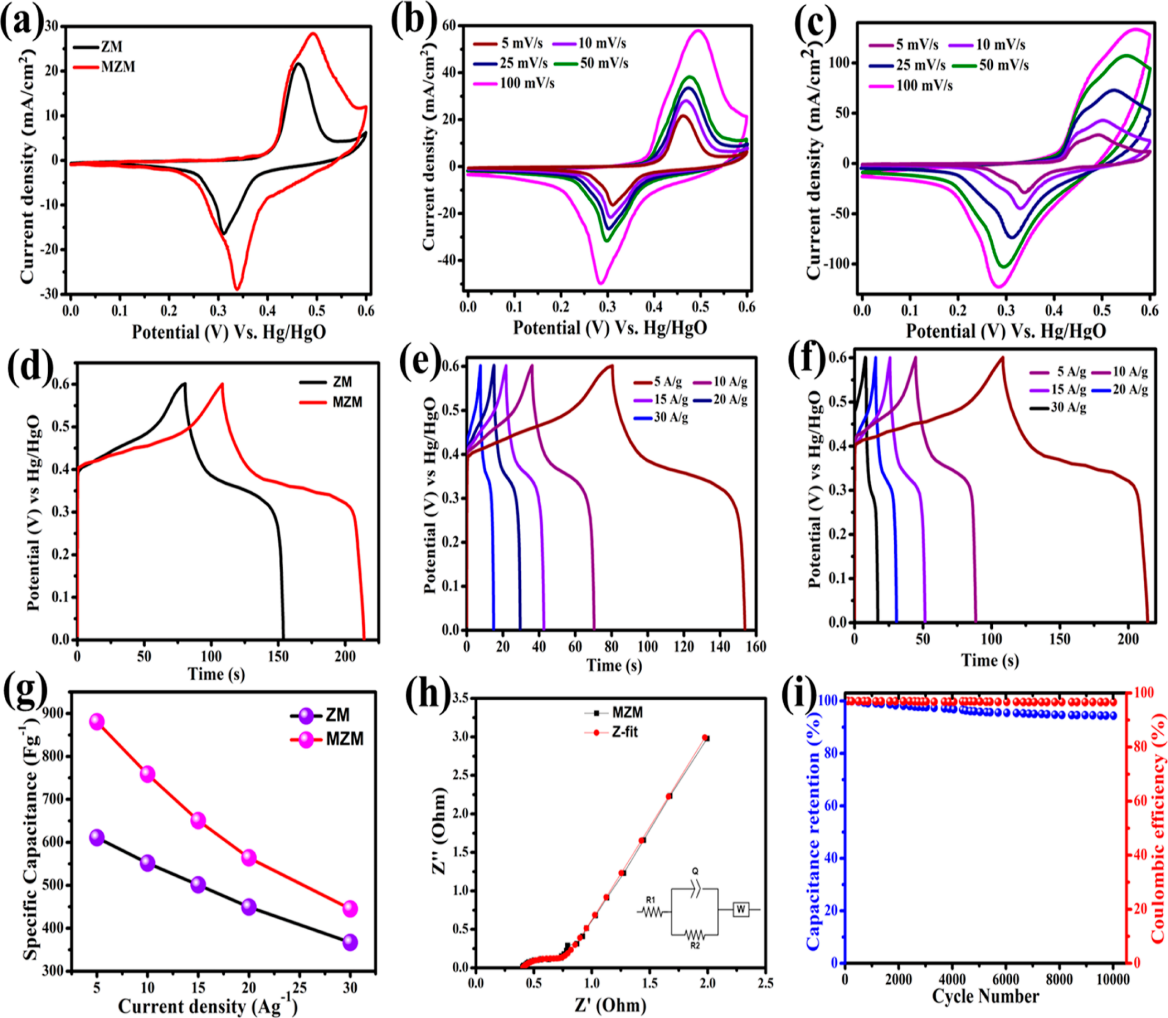
(a) Comparative CV curves of ZM and MZM electrodes at a constant current density of 5 mV s^–1^, (b) CV curves of the ZM electrode, and (c) MZM electrode at different scan rates (5, 10, 25, 50, and 100 mV s^–1^), (d) comparative GCD curves of ZM and MZM electrodes at a constant current density of 5 A g^–1^, (e) GCD curves of ZM electrode, and (f) MZM electrode at different current densities (5, 10, 15, 20, and 30 A g^–1^), (g) specific capacitance versus current density plot of ZM and MZM electrodes, (h) Nyquist plot of the MZM electrode fitted with an equivalent circuit, and (i) stability performance of the MZM electrode

A three-electrode cell was used to study the electrochemical properties of electrode materials where the Pt wire, Hg/HgO, and the prepared electrode were used as the counter, reference, and working electrodes, respectively, and the electrochemical tests were conducted in 3 M KOH. Figure 5a shows the CV curves of ZM and MZM electrodes measured in the potential range of 0 to 0.6 V vs Hg/HgO at a scan rate of 5 mV s^–1^. The non-linear curve and the sharp redox peaks indicate the faradaic pseudocapacitive behavior. A pair of prominent redox peaks about 0.31 and 0.46 V (vs Hg/HgO) can be seen, which was indicative of the transition between Zn(II) and Zn(III). These redox processes enable a faradaic charge storage mechanism in ZM electrodes, and similar charge storage behavior has been seen in other metal-BDC crystals in reported works.^53,54^ The intercalation and de-intercalation of the alkali metal ion K^+^ from the electrolyte with the free ZnO clusters in the electrode surface raise this set of strong faradaic redox peaks.^55,56^ The redox process is shown below^57,58^
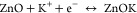
5

The unique morphology of MOFs with structural defects and easily accessible metal centers is helpful in the fast diffusion of K^+^ ions, facilitating a better interaction between the electrode and electrolyte.^59,60^ The CV curve of the MZM electrode exhibits a relatively larger integral area and a greater current density when compared to the ZM electrode which indicates a higher specific capacitance. It can be noted that the addition of α-MnO_2_ produced additional redox peaks around 0.35 and 0.49 V in the MZM electrode. This is due to the surface adsorption–desorption of K^+^ ions by α-MnO_2_ to store energy. This involves the conversion of Mn(III) to Mn(IV) and Mn(IV) to Mn(III) by a reversible surface redox reaction between Mn^4+^ and Mn^3+^ ions.^61−64^ The reaction mechanism is shown below
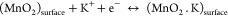
6
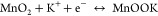
7

Figure 5b,c represents CV curves of ZM and MZM electrode materials, respectively, at various scan rates of 5, 10, 25, 50, and 100 mV s^–1^ within a potential range of 0–0.6 V. At higher scan rates, the integral area under the CV curve and current density increased, and the shape of the curve remained consistent, suggesting strong reversibility of electrode materials.^65^ It can be seen that the contour of CV curves of the electrodes is preserved even at high scan rates (100 mV/s), which guaranteed the ultra-fast rate kinetics of the working electrode’s faradaic process.^55^ Moreover, CV curves of pure α-MnO_2_ show clearly visible redox peaks at a lower scan rate of 5 mV/s (Figure S1a) and slightly change with higher scan rates (Figure S1b), which indicates that the process of energy storage by α-MnO_2_ was mainly associated with a redox mechanism and not the reaction between the Mn4^+^ ions and hydroxide in the electrolyte.^66^ Further, while comparing the CV curves of different quantities (10, 20, 30, and 40 mg) of MnO_2_-incorporated Zn-MOFs (Figure S6a), it can be seen that the integral area of MZM electrode material (20 mg) is larger than the M-10, M-30 and M-40, indicating the superior performance of MZM electrode materials.

The GCD measurements were carried out at varied current densities (5 to 30 A g^–1^) in the potential window range of 0 to 0.6 V. The GCD curves of ZM and MZM electrodes at a constant current density of 5 A/g are shown in Figure 5d, and the GCD plot of α-MnO_2_ is given in Figure S2c (see the Supporting Information). Due to redox activities that occur during the electrochemical charge and discharge processes, all the electrodes show a nonlinear-shaped GCD curve.^67^ This is consistent with the CV results. Notably, the MZM electrode displays a longer discharge duration and a larger integral area beneath the GCD curve than the ZM electrode, indicating improved electrochemical performance. Figure 5e,f shows the GCD curves of ZM and MZM electrodes, respectively, at different current densities (5–30 A g^–1^). Non-linear discharge curves at all current densities with the presence of a voltage plateau confirm the faradaic pseudocapacitive characteristics of the electrodes.^68^ A similar kind of discharge process can be seen in GCD curves of α-MnO_2_ (Figure S1d). This nature of non-linearity is attributed to voltage-dependent charge-transfer redox reactions.^69^ During the discharge, a sudden potential drop could be seen, which represents energy losses arising from the internal resistance of the setup.^70^ Moreover, while comparing GCD curves of different quantities (10, 20, 30, and 40 mg) of MnO_2_-incorporated Zn-MOFs (Figure S6b), it can be observed that the MZM electrode material demonstrated longer discharging time than the M-10 electrode material, which exhibited further longer discharge-time than M-30 and M-40 samples. This suggests that, in accordance with XRD results, optimal quantity of 20 mg of MnO_2_ incorporation improved the crystallinity of MOFs, which resulted in better electrochemical performance.

Figure 5g illustrates the plot between the calculated specific capacitances and current densities of ZM and MZM electrodes. Specific capacitance values of ZM and MZM electrodes were calculated using the GCD curves using eq 1. At a current density of 5 A g^–1^, the determined specific capacitance values of the α-MnO_2_, ZM, and MZM electrodes were 541.69, 610.83, and 880.58 F g^–1^, respectively. The specific capacitances of the MZM electrode were around 1.6 times greater than pure α-MnO_2_ and 1.4 times greater than the ZM electrode. At current densities of 5, 10, 15, 20, and 30 A g^–1^, computed specific capacitance values for the MZM electrode were 880.583, 732.166, 638.575, 508.66, and 445.00 F g^–1^, respectively. It is clear that specific capacitances of the electrodes decreased with increasing current densities, indicating the insufficient involvement of the electrode material in electrochemical processes at higher current densities. The reasons for the improvement in the electrochemical performance of the MZM electrode include (i) increased number of reactive sites, (ii) enhanced electronic conductivity, and (iii) improved redox activity arising from the inclusion of α-MnO_2_. EIS profiles in the frequency range of 0.1 Hz to 1 MHz were used to determine the electrode’s ion diffusion and electron-transfer properties. Figure 5h shows the Nyquist plot of MZM electrodes fitted with an equivalent circuit model. The obtained values of solution resistance (*R*_s_), charge-transfer resistance (*R*_CT_), and Warburg resistance (*W*) of the MZM electrodes are 0.404, 0.385, and 0.79 Ω, respectively. From the Nyquist plot of α-MnO_2_, ZM, and MZM with an equivalent circuit (Figure S3), the *R*_s_, *R*_ct_, and *W* values of α-MnO_2_ are 1.84, 0.137, 1.9, and for ZM, they were 0.712, 3.418, and 4.23 Ω, respectively. It can be seen that the *R*_s_ value of the MZM electrode (0.404 Ω) was much smaller when compared with α-MnO_2_ (1.84 Ω), and the *R*_ct_ value of the MZM electrode (0.385) was very small when compared with the ZM (3.418 Ω), which imparts that the conductivity of MZM electrodes was considerably improved. Moreover, the MZM electrode shows a more ideal straight line along with small Warburg resistance and thus contributes to the faster transfer of electrolyte ions into the electrode. Figure 5i displays the cyclic stability of the MZM electrode obtained at a high current density of 10 A g^–1^. The MZM electrode retained 94% of its initial capacitance over 10,000 GCD cycles with a coulombic efficiency of 99.4%, indicating the good stability of the electrode material. The XRD pattern and FESEM image of the MZM electrode material after cycling is shown in Figure S7a,b. From the XRD pattern (Figure S7a), it can be inferred that the characteristic peak (at θ = 9.8°) of MOFs is retained which demonstrates good reversibility. The significant reduction in the intensity of peaks may be due to the presence of CB and PVDF, which are added during electrode preparation. From the FESEM image (Figure S7b), it can be seen that the MOF almost maintains the same morphology after long cycles without any major changes. However, some of the particles seem to be aggregated due to the presence of PVDF and CB. The comparison of specific capacitance of our electrode material with previously reported material is tabulated (Table 1).

**Table 1:** Comparison of the Specific Capacitance of This Work with Other Previously Reported References

S. no.	electrode materials	electrolyte	specific capacitance (F g^–1^)	references
1	Mn-BDC MOF	1 M Na_2_SO_4_	177.9	(71)
2	ZIF-8/PANI	1 M H_2_SO_4_	236	(72)
3	UIO-66/rGO	6 M KOH	302	(73)
4	Ni-MOF(HU16)	6 M KOH	307	(74)
5	ZIF-67/PANI	3 M KCl	371	(75)
6	Cu-MOF/rGO	PVA–Na2SO4	385	(76)
7	ZIF-67@Mn-ZIF derived Co3O4@MnO2	1 M LiOH	413	(77)
8	ZIF-67/polypyrrole nanotubes	1 M Na_2_SO_4_	597.6	(78)
9	Ni-MOF-5/rGO	1 M KOH	758	(79)
10	MnO2@Zn-MOF	3 M KOH	880.58	present work

### 3.2 ASC Device

**Figure 6:**
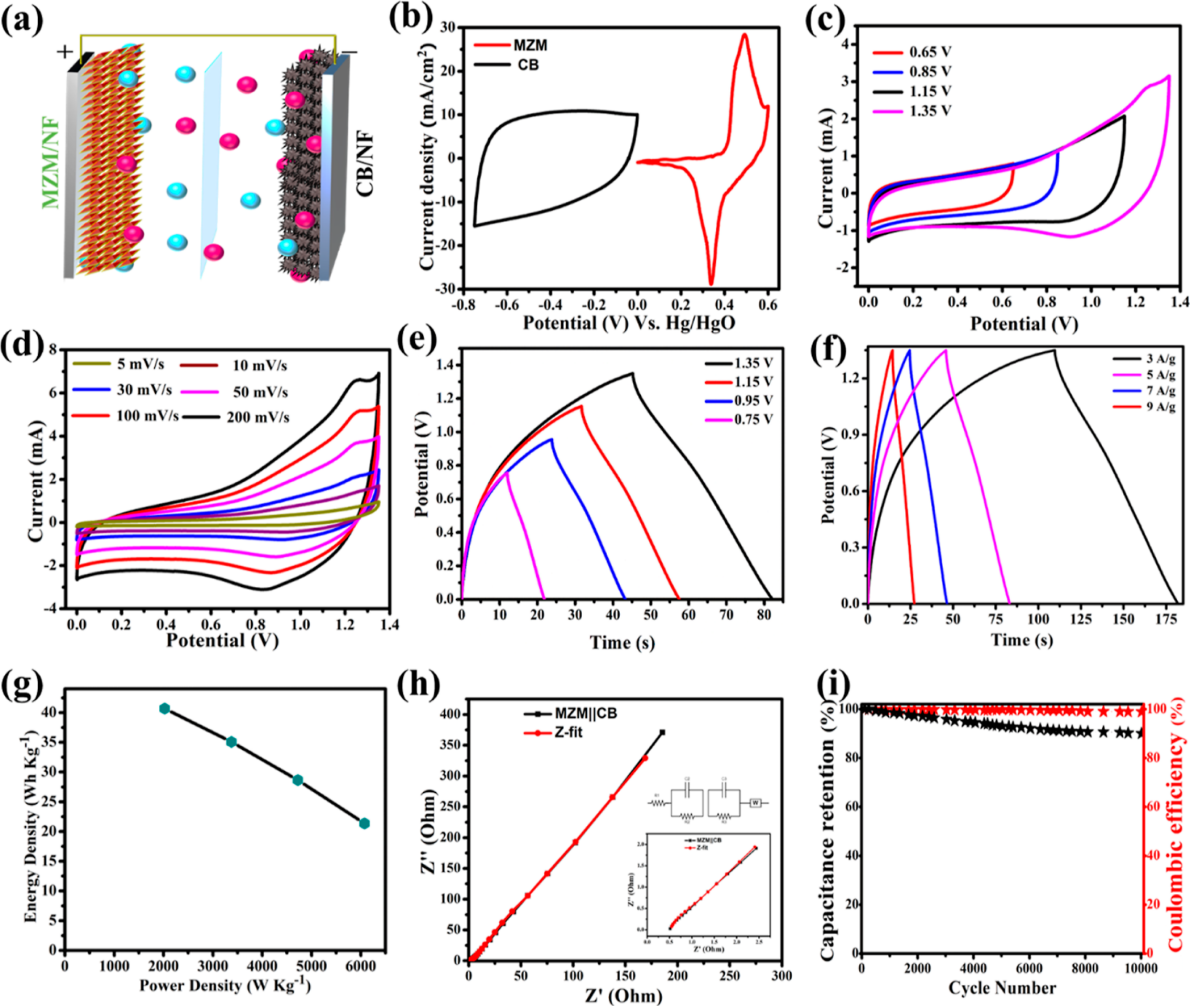
(a) Schematic representation of ASC, (b) CV curves of CB and MZM electrodes at 5 mV s^–1^ scan rate, (c) CV curves of ASC device in different potential windows at a constant scan rate of 5 mV s^–1^, (d) CV curves of ASC at various scan rates (5, 10, 25, 50, and 100 mV s^–1^) in a potential window of 0 to 1.35 V, (e) GCD curves of ASC in different operating potentials at a constant current density of 5 A g^–1^, (f) GCD curves of ASC at different current densities (3, 5, 7, and 9 A g^–1^) in a potential window of 0–1.35 V, (g) Ragone plot of ASC, (h) Nyquist plot of ASC device fitted with the equivalent circuit, and (i) stability performance of ASC.

In order to examine the practical performance of the prepared electrode, a non-aqueous ASC was made by employing the as-prepared MZM electrode and commercial CB as the positive and negative electrodes, respectively, in the PVA + KOH gel electrolyte, as shown in the schematic diagram in Figure 6a. The PVA + KOH gel electrolyte was made by mixing 5 g of KOH and 10 g of PVA in 150 mL of distilled water and stirring it at 90 °C continuously until a clear solution is formed, then it is dried at room temperature. Moreover, designing the ASCs requires careful consideration of the appropriate operating potential window (OPW) of the positive and negative electrodes.^80,81^

Consequently, the CV experiment was conducted at a scan rate of 5 mV s^–1^ in the three-electrode setup to assess the OPW of the positive and negative electrodes, as shown in Figure 6b. The determined OPW was 0 to 0.6 V for the MZM electrode and −0.75 to 0 V for the CB electrode. Therefore, in order to attain higher energy and power densities, the device should operate at a potential of 1.35 V. The CV curves for the assembled ASC measured in various OPWs at a fixed scan rate of 5 mV s^–1^ are shown in Figure 6c. As a result of combined electric-double layer capacitance of CB and redox-faradic behavior of MZM, all CV curves exhibit a quasi-rectangular shape when the OPW range was altered from 0.65 to 1.35 V.^82,83^ The CV curves of ASC in the potential range of 0–1.35 V at varied scan rates between 5 and 200 mV s^–1^ are shown in Figure 6d. Even at an increased scanning rate of 200 mV s^–1^, the CV curves maintained their shape, indicating good rate performance. The GCD measurement was carried out in various OPWs at a current density of 5 A g^–1^ in order to further corroborate the OPW of ASCs (Figure 6e). The sloping discharge pseudo-plateau is visible on all GCD curves, and this observation is in accordance with CV results. Additionally, the ASCs could operate up to 1.35 V without any alterations in GCD plots. Figure 6f shows the GCD plots of ASC at different current densities ranging from 3 to 9 A g^–1^. Specific capacitance values of the ASC at 3, 5, 7, and 9 A g^–1^ calculated using eq 1 were 160.72, 138.57, 113.17, and 84.37 F g^–1^, respectively. To compare the change in specific capacitance at different current densities, the values are represented as a bar graph in Figure S4 (Supporting Information). Energy and power densities are also important in determining the supercapacitor’s performance, which are calculated using eqs 2–4. A Ragone plot showing energy densities at various power densities of ASC is displayed in Figure 6g. At the lowest power density of 2024.98 W kg^–1^, the ASC displayed the highest energy density value of 40.64 W h kg^–1^. The EIS measurements were shown in Figure 6h as a Nyquist plot fitted with an equivalent circuit model, with an inset showing a closer view of lower values. The obtained solution resistance (*R*_s_), charge-transfer resistance (*R*_ct_), and Warburg impedance (*W*) values were 0.568, 0.519, and 2.477 Ω, respectively. This confirms that the device has a considerably low resistance thus promoting ion transfer for better conductivity and enhancing the overall performance of the device. Further, the ASC demonstrated long-term cyclic stability during 10,000 charge–discharge cycles at a constant current density of 9 A g^–1^ and maintained 90% of its original capacitance with a coulombic efficiency of 98% (Figure 6i). The comparison of energy density and power density of assembled ASCs with other devices from the literature is shown in Table.2.

**Table 2:** Comparison of the Power Density and Energy Density of Assembled ASC with Other Reported Devices

S. no.	name of the device	energy density (W h kg^–^^1^)	power density (W kg^–^^1^)	reference
1	Zn/Ni-MOF//RGO	30.51	800	(84)
2	Ag-MOF//AC	48.69	608.73	(85)
3	Ni-MOF@GO composite	30.7	388.5	(86)
4	symmetric Zn-MOF-rGO	7.1	400	(87)
5	NiCo-MOF//AC	49.4	562.5	(88)
6	MnO2@Ni-HHTP//AC	35.8	600	(24)
7	NiO@Ni-MOF//CNT	39.2	7000	(26)
8	MnO2@Zn-MOF//CB	40.68	2024.98	present work

The following reasons demonstrate the MZM||CB ASC’s enhanced electrochemical performance: (i) inclusion of α-MnO_2_ considerably improved the electrical conductivity, redox activity, and charge storage characteristics, (ii) commercial CB electrodes with higher electrical conductivity and chemical stability, (iii) MOF electrode material with unique morphology which binds to the electrode using CB facilitate quick ion movements and also offer a lot of active sites for electrochemical reactions, and (iv) large operational potential window of 0 to 1.35 V, which is equivalent of individual CB and MZM electrodes, which results in the high energy density.

## 4 Conclusions

In summary, α-MnO_2_ nanoflower-incorporated zinc-terephthalate MOF (MZM) was synthesized by the solution phase synthesis technique. The prepared electrode material exhibited a specific capacitance of 880.58 F g^–1^ at 5 A g^–1^ with a 94% capacitance retention after 10,000 cycles at 10 A g^–1^. The material showed superior electrochemical performance with enhanced conductivity, improved redox activity, and an increased number of reactive sites owing to the unique structure of MOFs and the addition of α-MnO_2._ Moreover, the ASC assembled using MZM (anode) and commercial CB (cathode) electrodes displayed a high energy density of 40.68 W h kg^–1^ at a power density of 2024.98 W kg^–1^ and shows a specific capacitance of 160 F g^–1^ at 3 A g^–1^. The device also demonstrated a good cycle stability of 90% retention of its initial capacitance after 10,000 cycles. Thus, α-MnO_2_ nanoflower-incorporated zinc-terephthalate electrode material is considered an attractive option with great potential for the future generation of energy storage devices.

## Supporting Information Available

High-resolution XPS spectrum, CV curves, Nyquist plots, specific capacitance versus current density plot of ASC, powder XRD patterns, comparative CV curves and GCD curves, XRD pattern and FE-SEM image, CV curves of CB electrode at different scan rates (5, 10, 25, 50, and 100 mV s^–1^), GCD curves of CB electrode at different current densities (5, 10, 15, 20, and 30 A g^–1^), and Nyquist plots of the CB electrode (PDF)

## References

[ref1] AL Shaqsi A. Z.; Sopian K.; Al-Hinai A. Review of Energy Storage Services, Applications, Limitations, and Benefits. Energy Rep. 2020, 6, 288–306. 10.1016/J.EGYR.2020.07.028.

[ref2] Simons S.; Schmitt J.; Tom B.; Bao H.; Pettinato B.; Pechulis M. Advanced concepts. Thermal, Mechanical, and Hybrid Chemical Energy Storage Systems 2021; Academic Press: London, 2021; pp 569–596.

[ref3] Qin H.; Liu P.; Chen C.; Cong H. P.; Yu S. H. A Multi-Responsive Healable Supercapacitor. Nat. Commun. 2021, 12, 4297 10.1038/s41467-021-24568-w. 34262049 PMC8280176

[ref4] Krishnamoorthy K.; Pazhamalai P.; Mariappan V. K.; Nardekar S. S.; Sahoo S.; Kim S. J. Probing the Energy Conversion Process in Piezoelectric-Driven Electrochemical Self-Charging Supercapacitor Power Cell Using Piezoelectrochemical Spectroscopy. Nat. Commun. 2020, 11, 2351 10.1038/s41467-020-15808-6. 32393749 PMC7214414

[ref5] Chen H.; Du X.; Liu X.; Wu R.; Li Y.; Xu C. Facile Growth of Nickel Foam-Supported MnCo2O4.5 Porous Nanowires as Binder-Free Electrodes for High-Performance Hybrid Supercapacitors. J. Energy Storage 2022, 50, 104297 10.1016/J.EST.2022.104297.

[ref6] Sun J.; Du X.; Wu R.; Zhang Y.; Xu C.; Chen H. Bundlelike CuCo2O4Microstructures Assembled with Ultrathin Nanosheets As Battery-Type Electrode Materials for High-Performance Hybrid Supercapacitors. ACS Appl. Energy Mater. 2020, 3, 8026–8037. 10.1021/ACSAEM.0C01458.

[ref7] Sun J.; Xu C.; Chen H. A Review on the Synthesis of CuCo2O4-Based Electrode Materials and Their Applications in Supercapacitors. J. Materiomics 2021, 7, 98–126. 10.1016/J.JMAT.2020.07.013.

[ref8] Liu Y.; Du X.; Li Y.; Bao E.; Ren X.; Chen H.; Tian X.; Xu C. Nanosheet-Assembled Porous MnCo2O4.5 Microflowers as Electrode Material for Hybrid Supercapacitors and Lithium-Ion Batteries. J. Colloid Interface Sci. 2022, 627, 815–826. 10.1016/J.JCIS.2022.07.105. 35901561

[ref9] Wu R.; Sun J.; Xu C.; Chen H. MgCo2O4-Based Electrode Materials for Electrochemical Energy Storage and Conversion: A Comprehensive Review. Sustainable Energy Fuels 2021, 5, 4807–4829. 10.1039/D1SE00909E.

[ref10] Takeuchi K.; Hayashi T.; Kim Y. A.; Fujisawa K.; Endo M. The State-of-the-Art Science and Applications of Carbon Nanotubes. Nanosyst.: Phys., Chem., Math. 2014, 5, 15–24.

[ref11] Huang S.; Zhu X.; Sarkar S.; Zhao Y. Challenges and Opportunities for Supercapacitors. APL Mater. 2019, 7, 100901 10.1063/1.5116146.

[ref12] Sculley J.; Yuan D.; Zhou H. C. The Current Status of Hydrogen Storage in Metal–Organic Frameworks. Energy Environ. Sci. 2011, 4, 2721–2735. 10.1039/C1EE01240A.

[ref13] Cui Y.; Xu H.; Yue Y.; Guo Z.; Yu J.; Chen Z.; Gao J.; Yang Y.; Qian G.; Chen B. A Luminescent Mixed-Lanthanide Metal-Organic Framework Thermometer. J. Am. Chem. Soc. 2012, 134, 3979–3982. 10.1021/JA2108036. 22352469

[ref14] Banerjee R.; Furukawa H.; Britt D.; Knobler C.; O’Keeffe M.; Yaghi O. M. Control of Pore Size and Functionality in Isoreticular Zeolitic Imidazolate Frameworks and Their Carbon Dioxide Selective Capture Properties. J. Am. Chem. Soc. 2009, 131, 3875–3877. 10.1021/JA809459E. 19292488

[ref15] Kong X.; Scott E.; Ding W.; Mason J. A.; Long J. R.; Reimer J. A. CO 2 Dynamics in a Metal-Organic Framework with Open Metal Sites. J. Am. Chem. Soc. 2012, 134, 14341–14344. 10.1021/JA306822P. 22908934

[ref16] Wang C.; Kim J.; Tang J.; Kim M.; Lim H.; Malgras V.; You J.; Xu Q.; Li J.; Yamauchi Y. New Strategies for Novel MOF-Derived Carbon Materials Based on Nanoarchitectures. Chem 2020, 6, 19–40. 10.1016/J.CHEMPR.2019.09.005.

[ref17] Patah A.; Kasim W.; Yuliarto B. Potential Application Zn-MOF/MnO2 Composite as Methanol Gas Sensor. Key Eng. Mater. 2019, 811, 113–119. 10.4028/www.scientific.net/kem.811.113.

[ref18] Baumann A. E.; Burns D. A.; Liu B.; Thoi V. S. Metal-Organic Framework Functionalization and Design Strategies for Advanced Electrochemical Energy Storage Devices. Commun. Chem. 2019, 2, 86 10.1038/s42004-019-0184-6.

[ref19] Sundriyal S.; Kaur H.; Bhardwaj S. K.; Mishra S.; Kim K. H.; Deep A. Metal-Organic Frameworks and Their Composites as Efficient Electrodes for Supercapacitor Applications. Coord. Chem. Rev. 2018, 369, 15–38. 10.1016/J.CCR.2018.04.018.

[ref20] Jadhav S.; Kalubarme R. S.; Terashima C.; Kale B. B.; Godbole V.; Fujishima A.; Gosavi S. W. Manganese Dioxide/ Reduced Graphene Oxide Composite an Electrode Material for High-Performance Solid State Supercapacitor. Electrochim. Acta 2019, 299, 34–44. 10.1016/J.ELECTACTA.2018.12.182.

[ref21] Wu D.; Xie X.; Zhang Y.; Zhang D.; Du W.; Zhang X.; Wang B. MnO2/Carbon Composites for Supercapacitor: Synthesis and Electrochemical Performance. Front. Mater. 2020, 7, 2 10.3389/FMATS.2020.00002.

[ref22] Zhang Y. Z.; Cheng T.; Wang Y.; Lai W. Y.; Pang H.; Huang W. A Simple Approach to Boost Capacitance: Flexible Supercapacitors Based on Manganese Oxides@MOFs via Chemically Induced In Situ Self-Transformation. Adv. Mater. 2016, 28, 5242–5248. 10.1002/ADMA.201600319. 27145232

[ref23] Chen Y.; Kang C.; Ma L.; Fu L.; Li G.; Hu Q.; Liu Q. MOF-Derived Fe2O3 Decorated with MnO2 Nanosheet Arrays as Anode for High Energy Density Hybrid Supercapacitor. Chem. Eng. J. 2021, 417, 129243 10.1016/J.CEJ.2021.129243.

[ref24] Duan H.; Zhao Z.; Lu J.; Hu W.; Zhang Y.; Li S.; Zhang M.; Zhu R.; Pang H. When Conductive MOFs Meet MnO2: High Electrochemical Energy Storage Performance in an Aqueous Asymmetric Supercapacitor. ACS Appl. Mater. Interfaces 2021, 13, 33083–33090. 10.1021/ACSAMI.1C08161. 34235934

[ref25] Ding Z.; Cheng Z.; Shi N.; Guo Z.; Ren Y.; Han M.; Chen M.; Xie L.; Huang W. Dual-Electroactive Metal–Organic Framework Nanosheets as Negative Electrode Materials for Supercapacitors. Chem. Eng. J. 2022, 450, 137193 10.1016/J.CEJ.2022.137193.

[ref26] Xiong S.; Jiang S.; Wang J.; Lin H.; Lin M.; Weng S.; Liu S.; Jiao Y.; Xu Y.; Chen J. A High-Performance Hybrid Supercapacitor with NiO Derived NiO@Ni-MOF Composite Electrodes. Electrochim. Acta 2020, 340, 135956 10.1016/J.ELECTACTA.2020.135956.

[ref27] Du Y.; Li G.; Zhao L.; Ye L.; Che C.; Liu X.; Liu H.; Yang X. Core-Shell MnO2Nanotubes@Nickel-Cobalt-Zinc Hydroxide Nanosheets for Supercapacitive Energy Storage. ACS Appl. Nano Mater. 2020, 3, 7462–7473. 10.1021/ACSANM.0C01062.

[ref28] Revathi C.; Kumar R. T. R. Electro Catalytic Properties of α, β, γ, ϵ - MnO2 and γ - MnOOH Nanoparticles: Role of Polymorphs on Enzyme Free H2O2 Sensing. Electroanalysis 2017, 29, 1481–1489. 10.1002/ELAN.201600608.

[ref29] Li Q.; Lu W.; Li Z.; Ning J.; Zhong Y.; Hu Y. Hierarchical MoS2/NiCo2S4@C Urchin-like Hollow Microspheres for Asymmetric Supercapacitors. Chem. Eng. J. 2020, 380, 122544 10.1016/J.CEJ.2019.122544.

[ref30] Subramani K.; Sudhan N.; Divya R.; Sathish M. All-Solid-State Asymmetric Supercapacitors Based on Cobalt Hexacyanoferrate-Derived CoS and Activated Carbon. RSC Adv. 2017, 7, 6648–6659. 10.1039/C6RA27331A.

[ref31] Krishnamoorthy K.; Kim S. J. Growth, Characterization and Electrochemical Properties of Hierarchical CuO Nanostructures for Supercapacitor Applications. Mater. Res. Bull. 2013, 48, 3136–3139. 10.1016/J.MATERRESBULL.2013.04.082.

[ref32] Lu W.; Li Y.; Yang M.; Jiang X.; Zhang Y.; Xing Y. Construction of Hierarchical Mn2O3@MnO2Core-Shell Nanofibers for Enhanced Performance Supercapacitor Electrodes. ACS Appl. Energy Mater. 2020, 3, 8190–8197. 10.1021/ACSAEM.0C00392.

[ref33] Su D.; Ahn H. J.; Wang G. Hydrothermal Synthesis of α-MnO2 and β-MnO2 Nanorods as High Capacity Cathode Materials for Sodium Ion Batteries. J. Mater. Chem. A 2013, 1, 4845–4850. 10.1039/C3TA00031A.

[ref34] Hoseini Rad M.; Ghasemzadeh M. A.; Sharif M. S. Multi-component synthesis of spiro [indoline-3, 4’-pyrrolo [3, 4-c] pyrazoles] using Zn (BDC) metal-organic frameworks as a green and efficient catalyst. Iran. Chem. Commun. 2019, 7, 390–397. 10.33945/SAMI/ECC.2019.4.3.

[ref35] Dutta R.; Rao M. N.; Kumar A. Investigation of Ionic Liquid Interaction with ZnBDC-Metal Organic Framework through Scanning EXAFS and Inelastic Neutron Scattering. Sci. Rep. 2019, 9, 14741 10.1038/s41598-019-51344-0. 31611583 PMC6791939

[ref36] Peng M. M.; Jeon U. J.; Ganesh M.; Aziz A.; Vinodh R.; Palanichamy M.; Jang H. T. Oxidation of Ethylbenzene Using Nickel Oxide Supported Metal Organic Framework Catalyst. Bull. Korean Chem. Soc. 2014, 35, 3213–3218. 10.5012/BKCS.2014.35.11.3213.

[ref37] Javed R.; Zia M.; Naz S.; Aisida S. O.; Ain N. u.; Ao Q. Role of Capping Agents in the Application of Nanoparticles in Biomedicine and Environmental Remediation: Recent Trends and Future Prospects. J. Nanobiotechnol. 2020, 18, 172 10.1186/S12951-020-00704-4. PMC7682049 33225973

[ref38] Han Y.; Liu M.; Li K.; Sun Q.; Song C.; Zhang G.; Zhang Z.; Guo X. Cu2O Mediated Synthesis of Metal-Organic Framework UiO-66 in Nanometer Scale. Cryst. Growth Des. 2017, 17, 685–692. 10.1021/ACS.CGD.6B01533.

[ref39] Zhu S.; Liu Y.; Huo Y.; Chen Y.; Qu Z.; Yu Y.; Wang Z.; Fan W.; Peng J.; Wang Z. Addition of MnO2 in Synthesis of Nano-Rod Erdite Promoted Tetracycline Adsorption. Sci. Rep. 2019, 9, 16906 10.1038/S41598-019-53420-X. 31729438 PMC6858339

[ref40] Chorbadzhiyska E.; Bardarov I.; Hubenova Y.; Mitov M. Graphite–Metal Oxide Composites as Potential Anodic Catalysts for Microbial Fuel Cells. Catalysts 2020, 10, 796 10.3390/CATAL10070796.

[ref41] Dikio E. D.; Farah A. M. Synthesis, characterization and comparative study of copper and zinc metal organic frameworks. Chem. Sci. Trans. 2013, 2, 1386–1394. 10.7598/cst2013.520.

[ref42] Yu W.; Liu T.; Cao S.; Wang C.; Chen C. Constructing MnO2/Single Crystalline ZnO Nanorod Hybrids with Enhanced Photocatalytic and Antibacterial Activity. J. Solid State Chem. 2016, 239, 131–138. 10.1016/J.JSSC.2016.04.027.

[ref43] Fang T. Y.; Zeng Y. Z.; Liu Y. C.; Yang W. D. High-Performance Asymmetric Supercapacitors Fabricated by Amorphous MnO2 on 3D-Ni Foam as Positive Electrodes in a Mixed Electrolyte. J. Mater. Sci.: Mater. Electron. 2020, 31, 7672–7682. 10.1007/S10854-020-03303-Z.

[ref44] Xiao K.; Li J. W.; Chen G. F.; Liu Z. Q.; Li N.; Su Y. Z. Amorphous MnO2 Supported on 3D-Ni Nanodendrites for Large Areal Capacitance Supercapacitors. Electrochim. Acta 2014, 149, 341–348. 10.1016/J.ELECTACTA.2014.10.117.

[ref45] Pastor-Ramírez C.; Ulloa R. Z.; Ramírez-Rosales D.; Vázquez-Lima H.; Hernández-Anzaldo S.; Reyes-Ortega Y. Tetramer Compound of Manganese Ions with Mixed Valence [Mn II Mn III Mn IV ] and Its Spatial, Electronic, Magnetic, and Theoretical Studies. Crystals 2018, 8, 447 10.3390/CRYST8120447.

[ref46] Liao F.; Han X.; Cheng D.; Zhang Y.; Han X.; Xu C.; Chen H. MnO2 Hierarchical Microspheres Assembled from Porous Nanoplates for High-Performance Supercapacitors. Ceram. Int. 2019, 45, 1058–1066. 10.1016/J.CERAMINT.2018.09.285.

[ref47] Xie G.; Liu X.; Li Q.; Lin H.; Li Y.; Nie M.; Qin L. The Evolution of α-MnO2 from Hollow Cubes to Hollow Spheres and Their Electrochemical Performance for Supercapacitors. J. Mater. Sci. 2017, 52, 10915–10926. 10.1007/S10853-017-1116-4.

[ref48] Wei Y.-p.; Zhang Y.-w.; Chen J. S.; Mao C.-j.; Jin B. K. An Electrochemiluminescence Biosensor for P53 Antibody Based on Zn-MOF/GO Nanocomposite and Ag+-DNA Amplification. Microchim. Acta 2020, 187, 1–9. 10.1007/S00604-020-04425-1. 32683571

[ref49] Chen H.; Bao E.; Du X.; Ren X.; Liu X.; Li Y.; Xu C. Advanced Hybrid Supercapacitors Assembled with High-Performance Porous MnCo2O4.5 Nanosheets as Battery-Type Cathode Materials. Colloids Surf., A 2023, 657, 130663 10.1016/J.COLSURFA.2022.130663.

[ref50] Bao E.; Ren X.; Wu R.; Liu X.; Chen H.; Li Y.; Xu C. Porous MgCo2O4 Nanoflakes Serve as Electrode Materials for Hybrid Supercapacitors with Excellent Performance. J. Colloid Interface Sci. 2022, 625, 925–935. 10.1016/J.JCIS.2022.06.098. 35777099

[ref51] Liao F.; Han X.; Cheng D.; Zhang Y.; Han X.; Xu C.; Chen H. MnO2 Hierarchical Microspheres Assembled from Porous Nanoplates for High-Performance Supercapacitors. Ceram. Int. 2019, 45, 1058–1066. 10.1016/J.CERAMINT.2018.09.285.

[ref52] Wang H.; Yin F.; Chen B.; Li G. Synthesis of an ε-MnO2/Metal–Organic-Framework Composite and Its Electrocatalysis towards Oxygen Reduction Reaction in an Alkaline Electrolyte. J. Mater. Chem. A 2015, 3, 16168–16176. 10.1039/C5TA02244D.

[ref53] Yang Y.; Fan X.; Zhu S.; Xu H.; Yang Y.; Fan X.; Zhu S.; Xu H. Study on Capacitance of Zn-Based Electrode in Redox Electrolyte System. J. Mater. Sci. Chem. Eng. 2020, 08, 35–43. 10.4236/MSCE.2020.81004.

[ref54] Sundriyal S.; Mishra S.; Deep A. Study of Manganese-1,4-Benzenedicarboxylate Metal Organic Framework Electrodes Based Solid State Symmetrical Supercapacitor. Energy Procedia 2019, 158, 5817–5824. 10.1016/J.EGYPRO.2019.01.546.

[ref55] Pradeeswari K.; Venkatesan A.; Pandi P.; Karthik K.; Hari Krishna K. v.; Mohan Kumar R. Study on the Electrochemical Performance of ZnO Nanoparticles Synthesized via Non-Aqueous Sol-Gel Route for Supercapacitor Applications. Mater. Res. Express 2019, 6, 105525 10.1088/2053-1591/AB3CAE.

[ref56] Chen Y. L.; Hu Z. A.; Chang Y. Q.; Wang H. W.; Zhang Z. Y.; Yang Y. Y.; Wu H. Y. Zinc Oxide/Reduced Graphene Oxide Composites and Electrochemical Capacitance Enhanced by Homogeneous Incorporation of Reduced Graphene Oxide Sheets in Zinc Oxide Matrix. J. Phys. Chem. C 2011, 115, 2563–2571. 10.1021/JP109597N.

[ref57] Hassan K.; Farzana R.; Sahajwalla V. In-Situ Fabrication of ZnO Thin Film Electrode Using Spent Zn–C Battery and Its Electrochemical Performance for Supercapacitance. SN Appl. Sci. 2019, 1, 302 10.1007/S42452-019-0302-1.

[ref58] Zhang Y.; Li H.; Pan L.; Lu T.; Sun Z. Capacitive Behavior of Graphene–ZnO Composite Film for Supercapacitors. J. Electroanal. Chem. 2009, 634, 68–71. 10.1016/J.JELECHEM.2009.07.010.

[ref59] Liao P. Q.; Shen J. Q.; Zhang J. P. Metal–Organic Frameworks for Electrocatalysis. Coord. Chem. Rev. 2018, 373, 22–48. 10.1016/J.CCR.2017.09.001.

[ref60] Yang H. M.; Song X. L.; Yang T. L.; Liang Z. H.; Fan C. M.; Hao X. G. Electrochemical Synthesis of Flower Shaped Morphology MOFs in an Ionic Liquid System and Their Electrocatalytic Application to the Hydrogen Evolution Reaction. RSC Adv. 2014, 4, 15720–15726. 10.1039/C3RA47744D.

[ref61] Risch M.; Stoerzinger K. A.; Han B.; Regier T. Z.; Peak D.; Sayed S. Y.; Wei C.; Xu Z.; Shao-Horn Y. Redox Processes of Manganese Oxide in Catalyzing Oxygen Evolution and Reduction: An in Situ Soft X-Ray Absorption Spectroscopy Study. J. Phys. Chem. C 2017, 121, 17682–17692. 10.1021/ACS.JPCC.7B05592.

[ref62] Zhao X.; Hou Y.; Wang Y.; Yang L.; Zhu L.; Cao R.; Sha Z. Prepared MnO2 with Different Crystal Forms as Electrode Materials for Supercapacitors: Experimental Research from Hydrothermal Crystallization Process to Electrochemical Performances. RSC Adv. 2017, 7, 40286–40294. 10.1039/C7RA06369E.

[ref63] Kumar N.; Guru Prasad K.; Sen A.; Maiyalagan T. Enhanced Pseudocapacitance from Finely Ordered Pristine α-MnO2 Nanorods at Favourably High Current Density Using Redox Additive. Appl. Surf. Sci. 2018, 449, 492–499. 10.1016/J.APSUSC.2018.01.025.

[ref64] Jayachandran M.; Rose A.; Maiyalagan T.; Poongodi N.; Vijayakumar T. Effect of Various Aqueous Electrolytes on the Electrochemical Performance of α-MnO2 Nanorods as Electrode Materials for Supercapacitor Application. Electrochim. Acta 2021, 366, 137412 10.1016/J.ELECTACTA.2020.137412.

[ref65] Agudosi E. S.; Abdullah E. C.; Numan A.; Mubarak N. M.; Aid S. R.; Benages-Vilau R.; Gómez-Romero P.; Khalid M.; Omar N. Fabrication of 3D Binder-Free Graphene NiO Electrode for Highly Stable Supercapattery. Sci. Rep. 2020, 10, 1–13. 10.1038/s41598-020-68067-2. 32641769 PMC7343816

[ref66] Fang T. Y.; Zeng Y. Z.; Liu Y. C.; Yang W. D. High-Performance Asymmetric Supercapacitors Fabricated by Amorphous MnO2 on 3D-Ni Foam as Positive Electrodes in a Mixed Electrolyte. J. Mater. Sci.: Mater. Electron. 2020, 31, 7672–7682. 10.1007/S10854-020-03303-Z.

[ref67] Wang H.; Zhu C.; Wu M.; Zheng F.; Gao Y.; Niu H. Synthesis of a Novel Double-Ligand Nickel Conductive Metal–Organic Framework Material and Its Electrochemical Characterization for Supercapacitors. J. Mater. Sci. 2021, 56, 2517–2527. 10.1007/S10853-020-05378-9.

[ref68] Hu L.; Chen W.; Xie X.; Liu N.; Yang Y.; Wu H.; Yao Y.; Pasta M.; Alshareef H. N.; Cui Y. Symmetrical MnO2-Carbon Nanotube-Textile Nanostructures for Wearable Pseudocapacitors with High Mass Loading. ACS Nano 2011, 5, 8904–8913. 10.1021/NN203085J. 21923135

[ref69] Gund G. S.; Dubal D. P.; Chodankar N. R.; Cho J. Y.; Gomez-Romero P.; Park C.; Lokhande C. D. Low-Cost Flexible Supercapacitors with High-Energy Density Based on Nanostructured MnO2 and Fe2O3 Thin Films Directly Fabricated onto Stainless Steel. Sci. Rep. 2015, 5, 12454 10.1038/srep12454. 26208144 PMC4513645

[ref70] Bhujun B.; Tan M. T. T.; Shanmugam A. S. Study of Mixed Ternary Transition Metal Ferrites as Potential Electrodes for Supercapacitor Applications. Results Phys. 2017, 7, 345–353. 10.1016/J.RINP.2016.04.010.

[ref71] Sundriyal S.; Mishra S.; Deep A. Study of Manganese-1,4-Benzenedicarboxylate Metal Organic Framework Electrodes Based Solid State Symmetrical Supercapacitor. Energy Procedia 2019, 158, 5817–5824. 10.1016/J.EGYPRO.2019.01.546.

[ref72] Salunkhe R. R.; Tang J.; Kobayashi N.; Kim J.; Ide Y.; Tominaka S.; Kim J. H.; Yamauchi Y. Ultrahigh Performance Supercapacitors Utilizing Core–Shell Nanoarchitectures from a Metal–Organic Framework-Derived Nanoporous Carbon and a Conducting Polymer. Chem. Sci. 2016, 7, 5704–5713. 10.1039/C6SC01429A. 30034710 PMC6022217

[ref73] Sundriyal S.; Shrivastav V.; Kaur H.; Mishra S.; Deep A. High-Performance Symmetrical Supercapacitor with a Combination of a ZIF-67/RGO Composite Electrode and a Redox Additive Electrolyte. ACS Omega 2018, 3, 17348–17358. 10.1021/acsomega.8b02065.s001. 31458344 PMC6643819

[ref74] He H.; Wang G.; Shen B.; Wang Y.; Lu Z.; Guo S.; Zhang J.; Yang L.; Jiang Q.; Xiao Z. Three Isostructural Zn/Ni Nitro-Containing Metal-Organic Frameworks for Supercapacitor. J. Solid State Chem. 2020, 288, 121375 10.1016/J.JSSC.2020.121375.

[ref75] Wang L.; Feng X.; Ren L.; Piao Q.; Zhong J.; Wang Y.; Li H.; Chen Y.; Wang B. Flexible Solid-State Supercapacitor Based on a Metal-Organic Framework Interwoven by Electrochemically-Deposited PANI. J. Am. Chem. Soc. 2015, 137, 4920–4923. 10.1021/JACS.5B01613. 25864960

[ref76] Srimuk P.; Luanwuthi S.; Krittayavathananon A.; Sawangphruk M. Solid-Type Supercapacitor of Reduced Graphene Oxide-Metal Organic Framework Composite Coated on Carbon Fiber Paper. Electrochim. Acta 2015, 157, 69–77. 10.1016/J.ELECTACTA.2015.01.082.

[ref77] Xu J.; Xu C.; Zhao Y.; Wu J.; Hu J. Hollow Co3O4@MnO2 Cubic Derived From ZIF-67@Mn-ZIF as Electrode Materials for Supercapacitors. Front. Chem. 2019, 7, 831 10.3389/FCHEM.2019.00831. 31921762 PMC6923731

[ref78] Xu X.; Tang J.; Qian H.; Hou S.; Bando Y.; Hossain M. S. A.; Pan L.; Yamauchi Y. Three-Dimensional Networked Metal-Organic Frameworks with Conductive Polypyrrole Tubes for Flexible Supercapacitors. ACS Appl. Mater. Interfaces 2017, 9, 38737–38744. 10.1021/ACSAMI.7B09944. 29082737

[ref79] Banerjee P. C.; Lobo D. E.; Middag R.; Ng W. K.; Shaibani M. E.; Majumder M. Electrochemical Capacitance of Ni-Doped Metal Organic Framework and Reduced Graphene Oxide Composites: More than the Sum of Its Parts. ACS Appl. Mater. Interfaces 2015, 7, 3655–3664. 10.1021/AM508119C. 25612667

[ref80] Balamurugan J.; Thanh T. D.; Kim N. H.; Lee J. H. Facile Synthesis of 3D Hierarchical N-Doped Graphene Nanosheet/Cobalt Encapsulated Carbon Nanotubes for High Energy Density Asymmetric Supercapacitors. J. Mater. Chem. A 2016, 4, 9555–9565. 10.1039/C6TA03132C.

[ref81] Balamurugan J.; Nguyen T. T.; Aravindan V.; Kim N. H.; Lee J. H. Flexible Solid-State Asymmetric Supercapacitors Based on Nitrogen-Doped Graphene Encapsulated Ternary Metal-Nitrides with Ultralong Cycle Life. Adv. Funct. Mater. 2018, 28, 1804663 10.1002/ADFM.201804663.

[ref82] Xu H.; Hu X.; Yang H.; Sun Y.; Hu C.; Huang Y. Flexible Asymmetric Micro-Supercapacitors Based on Bi2O3 and MnO2 Nanoflowers: Larger Areal Mass Promises Higher Energy Density. Adv. Energy Mater. 2015, 5, 1401882 10.1002/AENM.201401882.

[ref83] Zhao P.; Yao M.; Ren H.; Wang N.; Komarneni S. Nanocomposites of Hierarchical Ultrathin MnO2 Nanosheets/Hollow Carbon Nanofibers for High-Performance Asymmetric Supercapacitors. Appl. Surf. Sci. 2019, 463, 931–938. 10.1016/J.APSUSC.2018.09.041.

[ref84] Zhang X.; Sui Y.; Wei F.; Qi J.; Meng Q.; Ren Y.; He Y. Self-Supported 3D Layered Zinc/Nickel Metal-Organic-Framework with Enhanced Performance for Supercapacitors. J. Mater. Sci.: Mater. Electron. 2019, 30, 18101–18110. 10.1007/S10854-019-02163-6.

[ref85] Shalini S. S.; Balamurugan R.; Velmathi S.; Bose A. C. Systematic Investigation on the Electrochemical Performance of Pristine Silver Metal-Organic Framework as the Efficient Electrode Material for Supercapacitor Application. Energy Fuels 2022, 36, 7104–7114. 10.1021/ACS.ENERGYFUELS.2C01034.

[ref86] Sahoo R.; Ghosh S.; Chand S.; Chand Pal S.; Kuila T.; Das M. C. Highly Scalable and PH Stable 2D Ni-MOF-Based Composites for High Performance Supercapacitor. Composites, Part B 2022, 245, 110174 10.1016/J.COMPOSITESB.2022.110174.

[ref87] Thi Q. V.; Patil S. A.; Katkar P. K.; Rabani I.; Patil A. S.; Ryu J.; Kolekar G.; Tung N. T.; Sohn D. Electrochemical Performance of Zinc-Based Metal-Organic Framework with Reduced Graphene Oxide Nanocomposite Electrodes for Supercapacitors. Synth. Met. 2022, 290, 117155 10.1016/J.SYNTHMET.2022.117155.

[ref88] Wang Y.; Liu Y.; Wang H.; Liu W.; Li Y.; Zhang J.; Hou H.; Yang J. Ultrathin NiCo-MOF Nanosheets for High-Performance Supercapacitor Electrodes. ACS Appl. Energy Mater. 2019, 2, 2063–2071. 10.1021/ACSAEM.8B02128.

