# Supplementary material for: Incorporation of α-MnO2 Nanoflowers into Zinc-Terephthalate Metal–Organic Frameworks for High-Performance Asymmetric Supercapacitors
Source: ACS Omega. 2023 Feb 9;8(7):6982–93. doi: 10.1021/acsomega.2c07808 (PMC9948164; doi:10.1021/acsomega.2c07808)
Supplement: Supplementary file 1 — ao2c07808_si_001.pdf [file ao2c07808_si_001.pdf]

Supporting Information

Incorporation of α-MnO_2 nanoflowers into Zinc- terephthalate Metal-Organic Framework for High-Performance Asymmetric Supercapacitor

Balaji Chettiannan [a], Arun Kumar Srinivasan[a], Gowdhaman Arumugam [a], Shanavas Shajahan[b], Mohammad Abu Haija [c], and, Ramesh Rajendran [a]*

[a]*Department of Physics, Periyar University, Salem 636011, Tamil Nadu, India.*

[b]*Department of Chemistry, Khalifa University, P.O. Box, 127788 Abu Dhabi, United Arab Emirates*

[c]*Center for Catalysis and Separations, Khalifa University of Science and Technology, P.O. Box., 127788 Abu Dhabi, United Arab Emirates*

**Corresponding Author e-mail:* rameshphys@gmail.com, (*R. Ramesh*)

List of Contents in Supporting Information

Figure S1. High-Resolution XPS Spectrum of O 1s of (a) α-MnO_2, (b) ZM, and (c) MZM electrode materials

Figure S2. CV curves of α-MnO_2 (a) at fixed (5 mV^{-1}) scan rate, and (b) at different scan rates, and GCD curves of α-MnO_2 (c) at constant (5 Ag^{-1}) current density and (d) at variable current densities

Figure S3. Nyquist Plots of α-MnO_2, ZM, and MZM electrodes with the fitted circuit

Figure S4. Specific capacitance versus current density plot of ASC

Figure S5. Powder XRD patterns of various quantities of α-MnO_2 incorporated in Zn-MOF

Figure S6. Comparative CV curves (a) of MZM, M-10, M-30, and M-40 electrode materials at fixed (5 mV^{-1}) scan rate, and Comparative GCD curves (b) of MZM, M-10, M-30, and M-40 electrode materials at constant (5 Ag^{-1}) current density

Figure S7. XRD pattern and FE-SEM image of MZM electrode material after cycling

Figure S8. CV curves (a) of Carbon Black (CB) electrode at different scan rates (5 mV s^{-1}, 10 mV s^{-1}, 25 mV s^{-1}, 50 mV s^{-1}, 100 mV s^{-1}), GCD curves (b) of CB electrode at different current densities (5 A g^{-1}, 10 A g^{-1}, 15 A g^{-1}, 20 A g^{-1}, 30 A g^{-1}), Nyquist Plots of CB electrode

Electrochemical analysis of Carbon Black

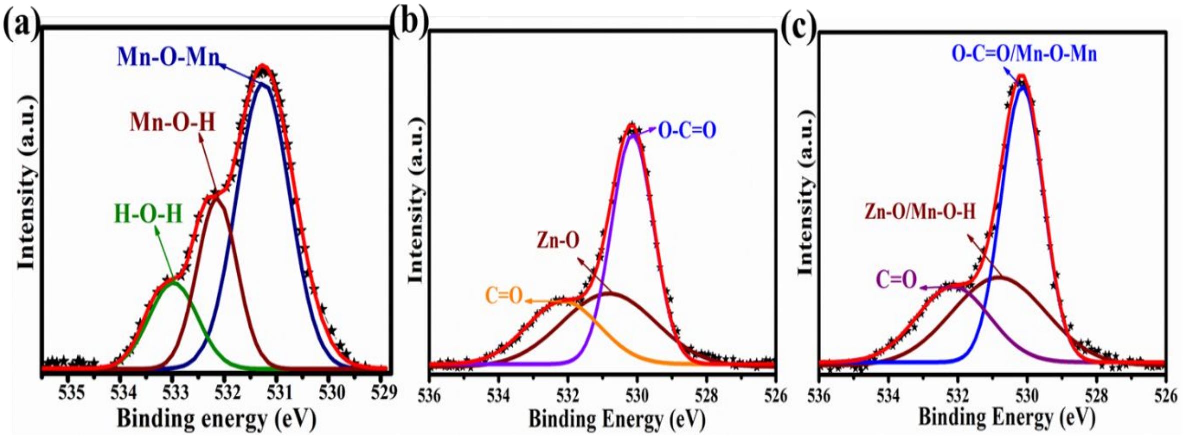

Figure S1. High-Resolution XPS Spectrum of O 1s of (a) α-MnO_2, (b) ZM, and (c) MZM electrode materials

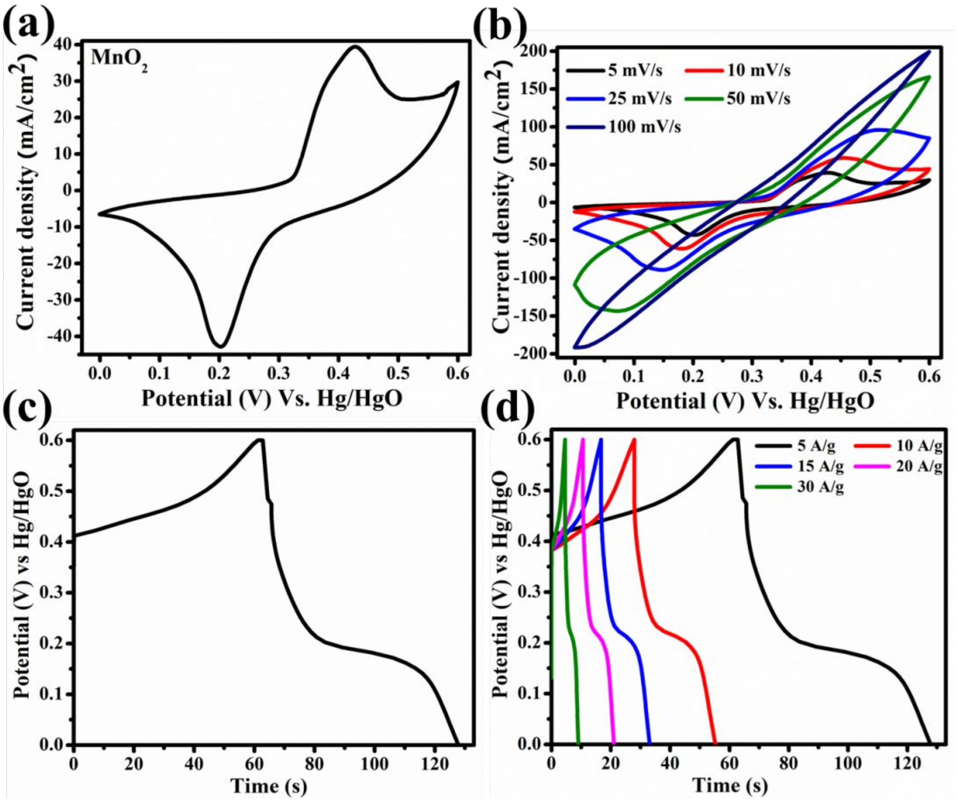

Figure S2. CV curves of α-MnO_2 (a) at fixed (5 mV^{-1}) scan rate, and (b) at different scan rates, and GCD curves of α-MnO_2 (c) at constant (5 Ag^{-1}) current density and (d) at variable current densities

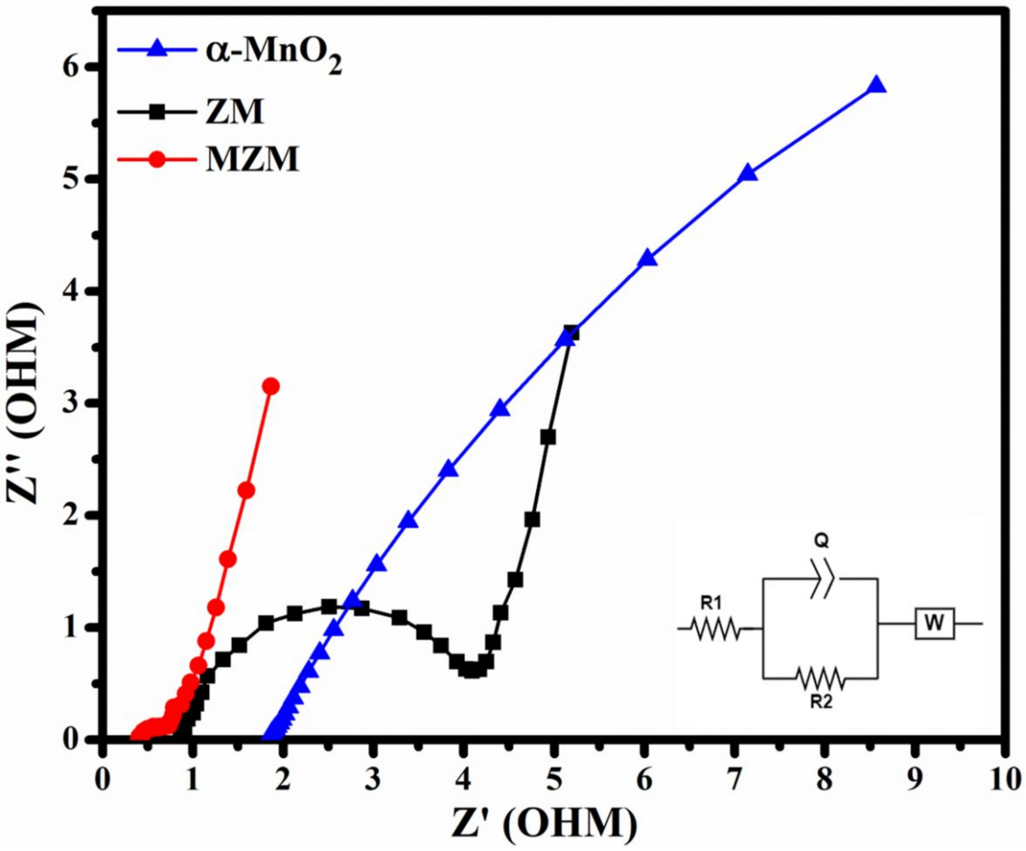

Figure S3. Nyquist Plots of α-MnO_2, ZM, and MZM electrodes with the fitted circuit

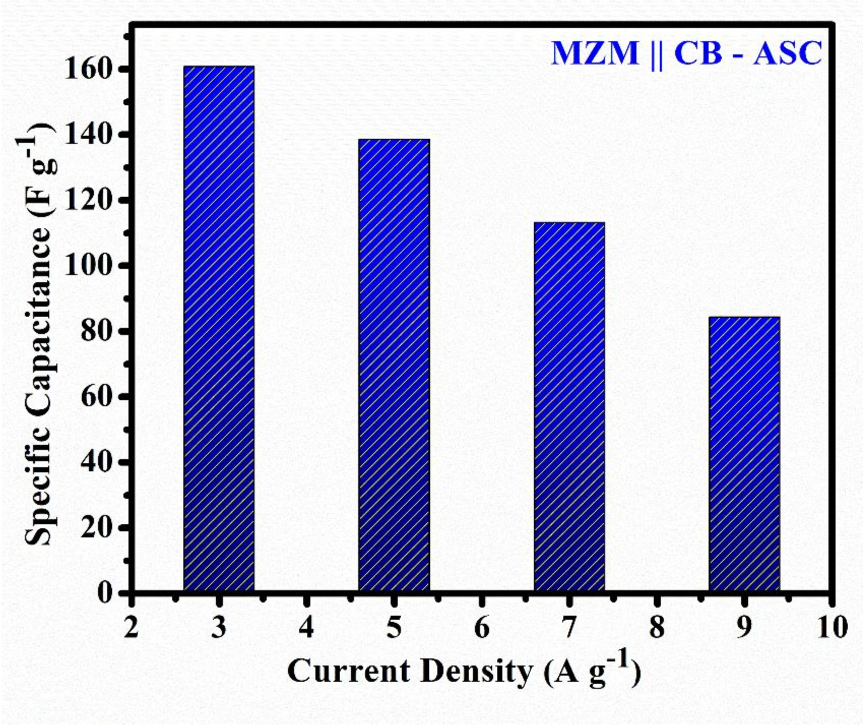

Figure S4. Specific capacitance versus current density plot of ASC

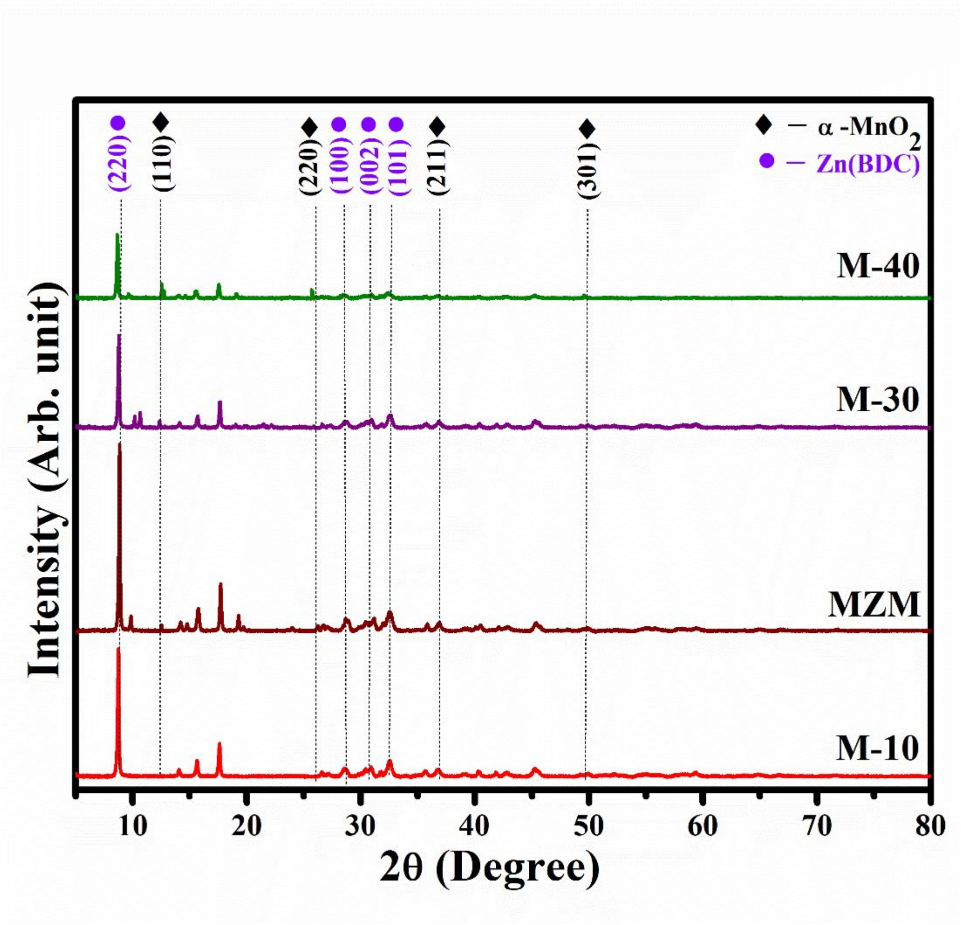

Figure S5. Powder XRD patterns of various quantities of α-MnO_2 incorporated in Zn-MOF

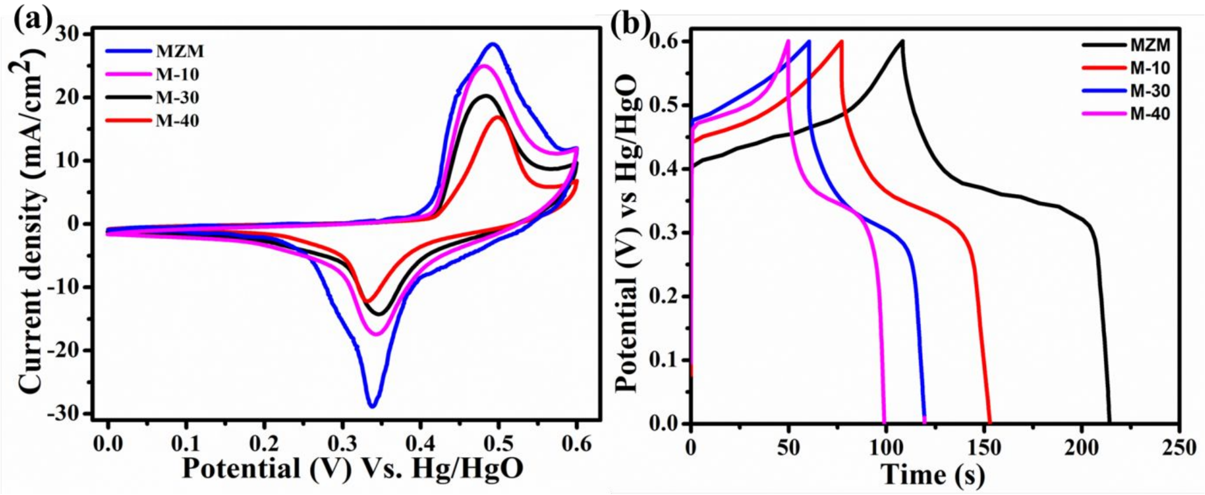

Figure S6. Comparative CV curves (a) of MZM, M-10, M-30, and M-40 electrode materials at fixed (5 mV^{-1}) scan rate, and Comparative GCD curves (b) of MZM, M-10, M-30, and M-40 electrode materials at constant (5 Ag^{-1}) current density

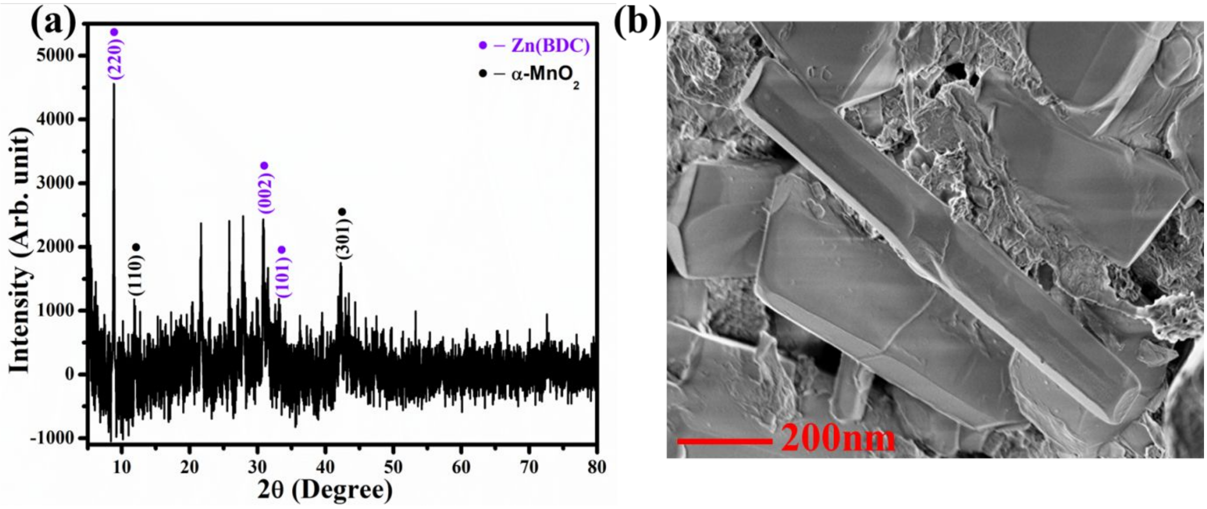

Figure S7. XRD pattern (a) and FE-SEM image (b) of MZM electrode material after cycling

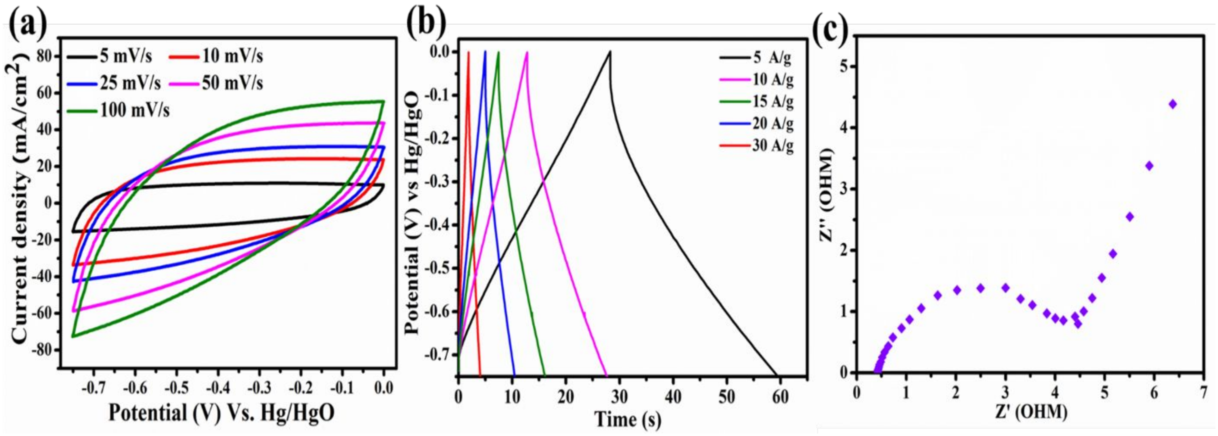

Figure S8. CV curves (a) of Carbon Black (CB) electrode at different scan rates (5 mV s^{-1}, 10 mV s^{-1}, 25 mV s^{-1}, 50 mV s^{-1}, 100 mV s^{-1}), GCD curves (b) of CB electrode at different current densities (5 A g^{-1}, 10 A g^{-1}, 15 A g^{-1}, 20 A g^{-1}, 30 A g^{-1}), Nyquist Plots of CB electrode (c)

Electrochemical analysis of Carbon Black:

A three-electrode cell was used to study the electrochemical properties of Carbon Black (CB), where Pt wire was used as the counter, Hg/HgO was used as the reference, and the Carbon Black electrode was used as the working electrode, and the electrochemical tests were conducted in 3M KOH electrolyte solution. Figure S8 (a) shows the CV curves of the CB electrode measured in the potential range of -0.75 to 0 V vs Hg/HgO in the scan rates of 5, 10, 25, 50, and 100 mV s^{-1}. The CV curves appear to be in rectangular shape without any redox peaks which confirms the dominance of the EDLC-type charge storage mechanism in the carbon black electrode. The GCD curves of the CB electrode at the current densities of 5, 10, 15, 20, and 30 A g^{-1} are shown in figure S8 (b). The GCD results show triangular-shaped curves, which is a characteristic behavior of EDLC. This is in accordance with the CV results. The specific capacitances of the CB were calculated from the GCD curves for different current densities. The calculated specific capacitance values at the current densities of 5, 10, 15, 20, and 30 A g^{-1} are 208.93, 198.8, 174, 145.06, and 87.20 F g^{-1}, respectively. Figure S8 (c) shows the Nyquist plot of the CB electrode at open circuit potential. It can be inferred that the R_s value of the CB electrode is 0.448 ohms, which indicates the high conductive nature of carbon black.
